# Prenatal exposure to perfluoroalkyl substances and thyroid hormone concentrations in cord plasma in a Chinese birth cohort

**DOI:** 10.1186/s12940-020-00679-7

**Published:** 2020-11-26

**Authors:** Hong Liang, Ziliang Wang, Maohua Miao, Youping Tian, Yan Zhou, Sheng Wen, Yao Chen, Xiaowei Sun, Wei Yuan

**Affiliations:** 1 Department of Social Medicine and Reproductive Epidemiology, National Health Commission Key Laboratory of Reproduction Regulation (Shanghai Institute of Planned Parenthood Research), Fudan University, #779 Lao Hu Min Road, Shanghai, 200032 China; 2 National Management Office of Neonatal Screening Project for CHD, Children’s Hospital of Fudan University, 399 Wanyuan Road, Shanghai, 201102 China; 3 National Reference Laboratory of Dioxin, Institute of Health Inspection and Detection, Hubei Provincial Academy of Preventive Medicine, Hubei Provincial Center for Disease Control and Prevention, #6 Zhuo Daoquan North Road, Wuhan, 430079 China

**Keywords:** Perfluoroalkyl substances, Prenatal exposure, Thyroid hormones, Cord blood, Bayesian kernel machine regression, Prospective cohort study

## Abstract

**Background:**

Evidence of associations between prenatal exposure to perfluoroalkyl substances (PFASs) and fetal thyroid hormones (THs) is controversial, and few studies have estimated the associations, while addressing the high correlations among multiple PFASs. We aimed to examine the associations between prenatal PFAS exposure and thyroid hormone concentrations in cord blood.

**Methods:**

A total of 300 mother-infant pairs from the Shanghai-Minhang Birth Cohort Study were included. We measured the concentrations of eight PFASs in maternal plasma samples collected at 12–16 gestational weeks, as well as those of total thyroxine (T4), free T4 (FT4), total triiodothyronine (T3), free T3 (FT3), and thyroid stimulating hormone (TSH) in cord plasma. We estimated the associations between maternal PFAS concentrations and TH concentrations using linear regression and Bayesian kernel machine regression (BKMR) models.

**Results:**

In BKMR models, higher PFAS mixture concentrations were associated with increased T3 concentrations, and there were suggestive associations with increased FT3 concentrations. For single-exposure effects in BKMR models, a change in PFDA, PFUdA, and PFOA concentrations from the 25th to 75th percentile was associated with a 0.04 (95%CrI: − 0.01, 0.09), 0.02 (95%CrI: − 0.03, 0.07), and 0.03 (95%CrI: − 0.001, 0.06) nmol/L increase in T3 concentrations, respectively. PFOA, PFNA, and PFDA were the predominant compounds in PFASs-FT3 associations, and the corresponding estimates were 0.11 (95% CrI: 0.02, 0.19), − 0.17 (95% CrI: − 0.28, − 0.07), and 0.12 (95% CrI: − 0.004, 0.24) pmol/L, respectively. A change in PFNA and PFOA concentrations from the 25th to 75th percentile was associated with a − 1.69 (95% CrI: − 2.98, − 0.41) μIU/mL decrease and a 1.51 (95% CrI: 0.48, 2.55) μIU/mL increase in TSH concentrations. The associations of PFOA and PFNA with T3/FT3 were more pronounced in boys, while those with TSH were more pronounced in girls.

**Conclusion:**

Our results suggest that prenatal exposure to multiple PFASs was associated with thyroid hormones in cord blood. However, individual PFAS had varied effects—differing in magnitude and direction—on fetal thyroid hormones.

**Supplementary Information:**

**Supplementary information** accompanies this paper at 10.1186/s12940-020-00679-7.

## Introduction

Perfluoroalkyl substances (PFASs) are synthetic fluorine-containing chemicals that have been extensively used in a range of industrial and commercial products during the past 60 years [1]. The general population is exposed to PFASs mainly through dietary intake of contaminated water and food, followed by indoor dust inhalation [2]. Certain PFASs, such as perfluorooctane sulfonate (PFOS), perfluorooctanoic acid (PFOA), perfluorohexane sulfonate (PFHxS), perfluorononanoic acid (PFNA), and perfluorodecanoic acid (PFDA), can be detected in 90% or more of the pregnant women in the US, Europe and Asia [3–7]. Since PFOS and PFOA were added to Annex B of the Stockholm Convention on Persistent Organic Pollutants in 2009, PFAS production has been restricted in many European countries and the US. However, other long-chain PFASs and new alternatives are being manufactured in increasing volumes in some countries, including China [8, 9]. PFASs are persistent in the environment due to their high resistance to degradation and long half-lives [1, 10]. Therefore, the adverse effects of PFAS exposure continue to pose a public health concern. Since PFASs can easily cross the placental barrier and expose the fetus [11, 12], there is significant concern regarding the adverse effects of in utero PFAS exposure.

Toxicological studies have demonstrated that prenatal PFAS treatment can disrupt thyroid function in pregnant rats and pups, characterized by hypothyroidism [13–16]. Thyroid hormones (THs) play a critical role in normal fetal and child growth and neurodevelopment. Alterations in maternal and neonatal thyroid functions are associated with neurodevelopmental and cognitive deficits in children [17, 18]. However, evidence regarding the effects of prenatal PFAS exposure on human fetal THs is inconsistent. Several studies have shown that maternal PFAS concentrations are associated with decreased fetal thyroxine (T4) and/or triiodothyronine (T3) concentrations accompanied by increased or unchanged thyroid stimulating hormone (TSH) concentrations [12, 19, 20]. Some studies have otherwise reported associations of cord PFASs with increased T4 and/or T3 concentrations accompanied by decreased or unchanged TSH [11, 21–23]. Most of the associations are observed in a sex-specific manner [19, 21–24]. In addition, most prior studies examined PFASs as individual chemicals although the actual exposure involves a mixture of highly correlated PFASs simultaneously, thus leaving the residual confounding effect of co-exposure uncontrolled. Multi-pollutant models are needed to adequately consider the complexity of multiple PFAS exposure, addressing highly correlated compounds and their potential interaction.

Our previous study based on the Shanghai-Minhang Birth Cohort Study in China reported that PFAS concentrations in pregnant women, especially PFOS and PFOA (geometric mean: 10.78 and 19.62 ng/ml, respectively), were much higher than those in the US and other Asian and European countries during a comparable time period [3]. Thus, in this study, we examined the associations between relatively higher concentrations of maternal plasma PFASs and thyroid hormone concentrations in cord blood, and evaluated the potential effect modification by infant sex. Moreover, we used the Bayesian kernel machine regression model, a recently developed multi-pollutant model, to assess the overall and single-exposure effects of the PFAS mixture on fetal THs.

## Methods

### Study design and participants

The present study was based on the Shanghai-Minhang Birth Cohort Study (S-MBCS), an ongoing prospective birth cohort study, which aims to determine the distributions of maternal environmental exposure and their effects on pregnant women and their children. Pregnant women at 12–16 gestational weeks were enrolled in the S-MBCS between April and December of 2012 during their first prenatal visit at the Maternal and Child Hospital of Minhang District in Shanghai. Participant recruitment criteria have been previously described in detail [3, 25]. Briefly, pregnant women were recruited if they had no history of major chronic diseases diagnosed by a physician, planned to deliver in this specific hospital, and were willing to attend the specified interviews during pregnancy and after delivery. At enrollment, pregnant women were interviewed face-to-face by trained interviewers using a structured questionnaire. Information about demographic characteristics and lifestyle, maternal parity, dietary intake, vitamin use, and disease history was obtained.

**Fig. 1:**
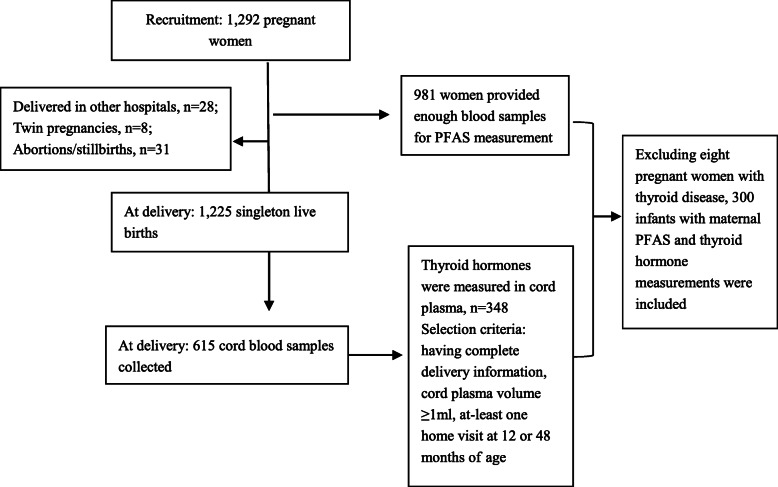
Flow chart of study population selection in the Shanghai-Minhang Birth Cohort Study, 2012

A total of 1292 pregnant women agreed to participate in the study (participation rate: 77.4%), 981 of whom had PFAS measurements from blood samples collected at enrollment. There were 1225 singleton live births at delivery, from which 615 cord blood samples were collected due to practical reasons. We further selected 348 cord blood samples for TH measurements under the conditions that the child had complete delivery information, had cord plasma volume ≥ 1 ml, and had at least one home visit at 12 or 48 months of age subject to limited funding. Finally, 308 mother-infant pairs had both maternal plasma PFAS concentrations and TH concentrations in cord blood measured. After excluding eight infants whose mothers reported prior or current diagnoses of thyroid-related disease/medication use at enrollment, 300 mother-infant pairs were included in the present study (Fig. 1).

The study protocol was approved by the Shanghai Institute of Planned Parenthood Research. All participants provided written informed consent for themselves and their children at enrollment and subsequent visits.

### Maternal PFAS measurements

One single blood sample was collected from each pregnant woman at 12–16 gestational weeks. After centrifugation, plasma samples were separated and stored at − 80 °C until shipment to the Center for Disease Control and Prevention in Hubei Province for PFAS measurements. The concentrations of eleven PFASs in maternal plasma were measured using high-performance liquid chromatography coupled with tandem mass spectrometry (HPLC-MS-MS; Agilent Technologies Inc., USA). A detailed protocol for sample preparation, measurement, and limits of detection (LODs) has been previously described [3]. In the present study, we included eight PFASs that were detected in more than 80% of plasma samples including PFHxS, PFOS, PFOA, PFNA, PFDA, perfluoroundecanoic acid (PFUdA), perfluorododecanoic acid (PFDoA), and perfluorotridecanoic acid (PFTrDA). Concentrations below the LOD were replaced by LOD/√2 in the analyses.

### Thyroid hormone assays in cord plasma

Cord blood sample was collected from an umbilical vein immediately after delivery. Plasma samples were separated and stored at − 80 °C. TH concentrations in cord plasma were measured at the clinical laboratory in the affiliated hospital of Shanghai Institute of Planned Parenthood Research. Concentrations of T3, free triiodothyronine (FT3), T4, free thyroxine (FT4), and TSH in cord plasma were measured by electrochemiluminescence immunoassay (ECLIA) with kits obtained from Roche Cobas e601 analyzer, as described previously [26]. The analytical sensitivities of T3, T4, FT3, FT4, and TSH were 0.3 nmol/L, 5.4 nmol/L, 0.4 pmol/L, 0.3 pmol/L, and 0.27 μIU/mL, respectively. The examiner was blinded to the infant’s exposure status at the time of assessment.

### Statistical analyses

We described and compared the demographic characteristics of the mother-infant pairs included and excluded from this study. We then described the distributions of maternal plasma PFAS concentrations and TH concentrations in cord plasma using percentiles and geometric means (GM). PFAS concentrations were natural log (ln) transformed to approximate the normal distribution for further analyses. The correlations between the pairs of ln-transformed PFAS concentrations and between the pairs of thyroid hormones were examined using Pearson correlation.

We first used the generalized additive model (GAM) to examine the non-linearity (*p*-value < 0.05) of the associations between maternal PFAS concentrations and THs in cord plasma, and some of the associations showed non-linearity (Supplemental Figure S1). We then conducted multiple linear regressions to examine the associations of THs with ln-transformed PFAS concentrations and PFAS tertiles, respectively. The variables that were risk factors for maternal PFAS or TH concentrations based on previous literature and our data were considered potential confounders, and a directed acyclic graph was used to identify the covariates (Supplemental Figure S2). The following variables were adjusted for in the final analyses: maternal age at delivery, pre-pregnancy body mass index (BMI: < 18.5, 18.5–23.9, and ≥ 24 kg/m^2^), education (high school or less, college or higher), parity (1, ≥ 2), gestational age (< 37 or ≥ 37 weeks), type of delivery (vaginal delivery, cesarean section), maternal passive smoking during pregnancy (yes, no), and maternal folic acid supplement during the first trimester (yes, no). Furthermore, paternal drinking during 3 months before pregnancy (yes, no) and infant sex (boy, girl) are strong predictors and they were included in the models to reduce imprecision. The information on maternal delivery date (to calculate age at delivery), type of delivery, gestational age, and infant sex was retrieved from medical birth records, while other information including maternal birth date, pre-pregnant weight and height, education, passive smoking during pregnancy, maternal folic acid supplement, and paternal drinking 3 months before pregnancy was obtained from the structured questionnaire. Maternal folic acid supplement was defined as taking the folic acid tablet regularly once a day. Paternal drinking was defined as paternal consumption of alcohol at least once a week in the last 3 months before conception and the information was reported by pregnant women at recruitment.

We further conducted a Bayesian kernel machine regression (BKMR) model, an approach for estimating the health effects of mixture exposure, to explore the overall effects of prenatal exposure to eight PFASs (ln-transformed concentrations) on each fetal TH, and to identify important PFASs in association with each TH, while adjusting for the same covariates as in the linear regression models. In addition, BKMR can assess potential interactions among multiple exposures. BKMR combines Bayesian and statistical learning methods to iteratively regress a response variable on a nonparametric term of exposure mixture components, through a Gaussian kernel function, that allows for non-linear and non-additive effects. Further, a hierarchical variable selection procedure within BKMR has been developed to account for the structure of the mixture and systematically handle highly correlated exposure [27, 28]. We presented the graphical output generated by BKMR to show the overall and single-exposure effects of the PFAS mixture on each TH. Figures of the overall effect show the point estimates and their 95% credible intervals (95% CrI) for the difference in each TH concentration holding concentrations of all eight PFASs at various quantiles (ranging from 0.25 to 0.75), as compared to fixing all PFAS concentrations at their 25th percentile. Figures of the single-exposure effect show the specific point estimates and their 95% CrIs for the difference in each TH concentration for a change in individual PFAS concentrations between the 25th and 75th percentile, when holding the other PFASs at their 25th, 50th, and 75th percentiles, respectively. The relative importance of each PFAS was assessed using posterior inclusion probabilities (PIPs) of > 0.5.

In the study, BKMR models were run in all participants, and then the analyses were stratified by infant sex due to its potential effect modification. We also stratified the analyses by type of delivery since it can influence FT3 and TSH concentrations [29]. To examine whether the associations between the PFAS mixture and THs were robust to the inclusion of additional co-exposed endocrine disrupting chemicals (EDCs), we fitted BKMR models by further including maternal urine bisphenol A (BPA) at recruitment and the sum of five polybrominated diphenyl ethers (Sum_5_PBDEs: BDE-28, − 47, − 99, − 100, and − 153) in cord blood as co-exposures. BPA and PBDE measurements have been introduced previously [26, 30]. We did not measure maternal iodine status, which is an important determinant of neonatal thyroid function. However, we collected the information on fish intake (iodine-rich food), and further adjusted for fish intake during the first trimester (≥3 days/week, 1–2 days/week, and < 1 day/week) in BKMR models to examine the robustness of the associations.

We used R software version 3.6.2 (R Development Core Team) to conduct GAMs and BKMR analyses, and used SAS version 9.4 (SAS Institute Inc., Cary, NC, USA) to perform other statistical analyses. The statistical analyses were two-sided, with *p*-value < 0.05 considered statistically significant.

## Results

The characteristics of the included and excluded mother-infant pairs are presented in Table 1. The included mothers had a mean age of 27.5 years, most of whom were nulliparous (87.0%), had normal pre-pregnant BMI (73.9%), and attained college or higher education (77.3%). About one-third of the included mothers reported a per capita household income of more than 8000 CNY/month, and about 43% experienced passive smoking before conception. Among the included infants, 55.6% were male, and 48.3% were born via cesarean section. Only 1.7% of infants were born preterm. The included mother-infant pairs had similar characteristics to those excluded, except for a lower percentage of preterm births (Table 1).

**Table 1:** Characteristics of mother-infant pairs included and excluded in the study

Variables^a^	Participants included (*N* = 300) n (%)	Participants excluded (*N* = 925) n (%)	*P*-value of Student’s t-test or Chi-square test
Maternal age at birth (years, mean ± SD)	27.5 ± 3.5	27.9 ± 3.4	0.0592
Maternal pre-pregnant BMI (kg/m^2^)			
< 18.5	63 (21.4)	181 (19.9)	0.8633
18.5–24.9	218 (73.9)	683 (75.1)	
≥ 25.0	14 (4.7)	45 (5.0)	
Parity			
Nulliparous	261 (87.0)	767 (83.7)	0.1744
Multiparous	39 (13.0)	149 (16.3)	
Maternal education			
High school or less	68 (22.7)	226 (24.5)	0.5460
College or higher	231 (77.3)	698 (75.5)	
Per capita household income (CNY/month)			
< 4000	66 (22.1)	187 (20.5)	0.2290
4000–8000	130 (43.5)	359 (39.5)	
> 8000	103 (34.4)	364 (40.0)	
Maternal passive smoking before conception			
No	171 (57.2)	556 (60.3)	0.3406
Yes	128 (42.8)	366 (39.7)	
Gestational age (weeks)			
≥ 37	295 (98.3)	884 (95.7)	0.0332
< 37	5 (1.7)	40 (4.3)	
Type of delivery			
Vaginal	154 (51.7)	368 (46.5)	0.1246
Cesarean section	144 (48.3)	424 (53.5)	
Child’s sex			
Male	167 (55.6)	500 (54.2)	0.6642
Female	133 (44.4)	422 (45.8)	

^a^*SD* Standard deviation, *BMI* Body mass index, *CNY* Chinese Yuan

PFOA was the dominant PFAS with the highest concentrations (GM: 19.41 ng/mL), followed by PFOS (GM: 10.77 ng/mL). GMs of PFHxS, PFDA, PFNA, PFUdA, PFDoA, and PFTrDA were 2.66, 2.15, 1.82, 1.61, 0.09, and 0.09 ng/mL, respectively, (Table 2). The distributions of eight PFASs in the included participants were comparable with those excluded (Table S1). As in our previous study, the ln-transformed PFAS concentrations were moderately to highly correlated with each other (Pearson correlation coefficients [r] = 0.33–0.89), except for the PFHxS-other compounds and PFOA-PFDoA/PFTrDA relationships (Supplemental Table S2). The GMs of cord blood T3, FT3, T4, FT4, and TSH concentrations were 0.85 nmol/L, 1.77 pmol/L, 90.63 nmol/L, 14.1 pmol/L, and 6.49 μIU/mL, respectively (Table 2). The correlations between the TH hormones were stronger between T4 and FT4 (*r* = 0.82) and between T3 and FT3 (*r* = 0.79). There were moderate correlations between FT3 and T4/FT4 (*r* = 0.44/0.49) and between TSH and T4/FT4 (*r* = 0.39/0.24). There was no correlation between other pairs of TH hormones (Supplemental Table S3). Infants born via vaginal delivery had statistically significantly higher TSH concentrations but lower FT3 and FT4 concentrations than those born via cesarean section (Supplemental Table S4).

**Table 2:** Descriptive statistics of PFAS concentrations in maternal plasma and thyroid hormone concentrations in cord plasma

	>LOD (%)	Percentiles					GM (SD)
		5th	25th	50th	75th	95th	
PFAS (*N* = 300, ng/mL)							
PFHxS	100	1.29	2.04	2.67	3.40	5.48	2.66 (1.51)
PFOS	100	4.04	7.34	10.49	16.30	26.47	10.77 (1.80)
PFOA	100	9.17	14.49	19.38	26.80	38.00	19.41 (1.56)
PFNA	100	0.84	1.38	1.80	2.51	3.88	1.82 (1.60)
PFDA	100	0.75	1.41	2.15	3.28	6.30	2.15 (1.91)
PFUdA	100	0.54	1.04	1.68	2.55	4.38	1.61 (1.95)
PFDoA	91.04	0	0.05	0.11	0.20	0.39	0.09 (3.09)
PFTrDA	88.93	0	0.04	0.11	0.18	0.37	0.09 (2.95)
Thyroid hormones (*N* = 300)							
T3 (nmol/L)	100	0.67	0.77	0.84	0.94	1.10	0.85 (1.18)
FT3 (pmol/L)	98	1.34	1.53	1.76	2.00	2.42	1.77 (1.21)
T4 (nmol/L)	100	49.30	77.67	93.10	109.90	143.65	90.63 (1.36)
FT4 (pmol/L)	100	11.08	12.82	14.14	15.39	17.81	14.10 (1.15)
TSH (μIU/mL)	100	2.24	4.32	6.50	9.72	18.97	6.49 (1.87)

*LOD* Limit of detection, *GM* Geometric mean, *SD* Standard deviation, *PFHxS* Perfluorohexane sulfonic acid, *PFOS* Perfluorooctane sulfonate, *PFOA* Perfluorooctanoic acid, *PFNA* Perfluorononanoic acid, *PFDA* Perfluorodecanoic acid, *PFUdA* Perfluoroundecanoic acid, *PFDoA* Perfluorododecanoic acid, *PFTrDA* Perfluorotridecanoic acid, *PFTeDA* Perfluorotetradecanoic acid, *PFHxDA* Perfluorohexadecanoic acid, *PFDS* Perfluorodecane sulfonate; T_3_, total triiodothyronine, *T*_*4*_ Total thyroxine, *FT*_*3*_ Free triiodothyronine, *FT*_*4*_ Free thyroxin, *TSH* Thyroid stimulating hormone

In linear regression models, ln-transformed concentrations of PFDoA, PFUdA, PFOS, PFNA, PFDA, and PFOA were associated with increased T3 concentrations [0.02 (95% confidence interval [CI]: 0.00, 0.04) to 0.07 (95% CI: 0.02, 0.11) nmol/L] in cord plasma after adjusting for covariates. Meanwhile, per ln-unit increase in PFOS, PFOA, and PFDA concentrations was associated with a 0.09 (95%CI: 0.02, 0.16), 0.13 (95%CI: 0.03, 0.23), and 0.08 (95%CI: 0.01, 0.15) pmol/L increase in FT3 concentrations, respectively, (Table 3). Ln-transformed concentrations of PFNA, PFDA, and PFUdA were associated with decreased T4 [− 7.81 (95%CI: − 14.38, − 1.24), − 5.07 (95%CI: − 9.78, − 0.37), and − 4.90 (95%CI: − 9.44, − 0.35) nmol/L, respectively] and TSH concentrations [− 1.61 (95% CI: − 2.90, − 0.31), − 0.96 (95% CI: − 1.89, − 0.03), and − 1.06 (95%CI: − 1.95, − 0.16) μIU/mL, respectively]. No association between PFASs and FT4 concentrations was observed (Table 3). The linear regression models using categorized PFAS concentrations showed similar results to those in the analyses above in general (Supplemental Table S5). However, the associations of PFHxS in middle tertile with increased T3, of PFDoA in middle tertile with increased FT3, of PFOA in middle tertile with decreased T4/FT4, and of PFTrDA in high tertile with decreased TSH, were observed.

**Table 3:** Associations between maternal plasma PFAS concentrations (ng/mL) and thyroid hormones in cord plasma by linear regression models

PFAS	T3 (nmol/L)		FT3 (pmol/L)		T4 (nmol/L)		FT4(pmol/L)		TSH (uIU/mL)	
	Crude β (95% CI)	Adjust β (95% CI)	Crude β (95% CI)	Adjust β (95% CI)	Crude β (95% CI)	Adjust β (95% CI)	Crude β (95% CI)	Adjust β (95% CI)	Crude β (95% CI)	Adjust β (95% CI)
PFHxS	0.00 (−0.05, 0.04)	0.00 (− 0.05, 0.04)	0.01 (− 0.10, 0.12)	0.02 (− 0.08, 0.13)	−1.70 (−9.10, 5.70)	− 0.59 (−7.94, 6.76)	−0.35 (− 0.89, 0.20)	− 0.32 (− 0.87, 0.22)	0.07 (− 1.45, 1.59)	0.43 (− 1.02, 1.88)
PFOS	0.05 (0.02, 0.08)*	0.05 (0.02, 0.08) *	0.07 (0.00, 0.15) *	0.09 (0.02, 0.16) *	−4.49 (− 9.68, 0.70)	−3.64 (−8.85, 1.56)	0.04 (− 0.34, 0.43)	0.06 (− 0.33, 0.45)	− 1.06 (−2.12, 0.00)	−0.71 (− 1.74, 0.31)
PFOA	0.06 (0.02, 0.11) *	0.07 (0.02, 0.11) *	0.13 (0.03, 0.23) *	0.13 (0.03, 0.23) *	−2.56 (− 9.48, 4.36)	− 1.18 (− 8.14, 5.79)	0.03 (− 0.48, 0.54)	0.08 (− 0.44, 0.59)	0.37 (− 1.05, 1.79)	0.86 (− 0.50, 2.23)
PFNA	0.04 (0.01, 0.08) *	0.05 (0.01, 0.09) *	0.02 (− 0.08, 0.11)	0.01 (− 0.08, 0.10)	−7.93 (− 14.43, − 1.42) *	−7.81 (− 14.38, − 1.24) *	− 0.34 (− 0.82, 0.14)	− 0.39 (− 0.88, 0.10)	−1.86 (− 3.20, − 0.53) *	−1.61 (− 2.90, − 0.31) *
PFDA	0.06 (0.03, 0.08) *	0.06 (0.03, 0.09) *	0.07 (0.01, 0.14) *	0.08 (0.01, 0.15) *	−5.40 (−10.09, − 0.71) *	−5.07 (− 9.78, − 0.37) *	− 0.10 (− 0.45, 0.25)	−0.10 (− 0.45, 0.26)	−1.07 (− 2.03, − 0.11) *	−0.96 (− 1.89, − 0.03) *
PFUdA	0.05 (0.02, 0.08) *	0.05 (0.03, 0.08) *	0.05 (− 0.01, 0.12)	0.06 (− 0.01, 0.12)	− 5.09 (− 9.66, − 0.53) *	−4.90 (− 9.44, − 0.35) *	−0.11 (− 0.45, 0.23)	−0.11 (− 0.45, 0.23)	− 1.11 (− 2.04, − 0.17) *	−1.06 (− 1.95, − 0.16) *
PFDoA	0.02 (0.00, 0.03) *	0.02 (0.00, 0.04) *	0.01 (−0.03, 0.05)	0.01 (− 0.03, 0.05)	−1.16 (− 3.88, 1.56)	−1.42 (− 4.15, 1.30)	0.03 (− 0.17, 0.23)	0.00 (− 0.20, 0.20)	−0.42 (− 0.98, 0.13)	−0.43 (− 0.97, 0.10)
PFTrDA	0.01 (− 0.01, 0.03)	0.01 (− 0.01, 0.03)	0 .00 (− 0.04, 0.04)	0.01 (− 0.03, 0.05)	− 2.37 (− 5.20, 0.47)	− 1.70 (− 4.54, 1.15)	−0.09 (− 0.30, 0.11)	−0.08 (− 0.29, 0.13)	−0.61 (− 1.19, − 0.03) *	−0.43 (− 0.99, 0.13)

PFAS concentrations were ln-transformed in the models; adjusted for maternal age at delivery, pre-pregnancy BMI, education, parity, gestational age, delivery type, infant’ sex, maternal passive smoking during pregnancy, maternal folic acid supplement, and paternal drinking during 3 months before pregnancy

*P < 0.05

**Fig. 2:**
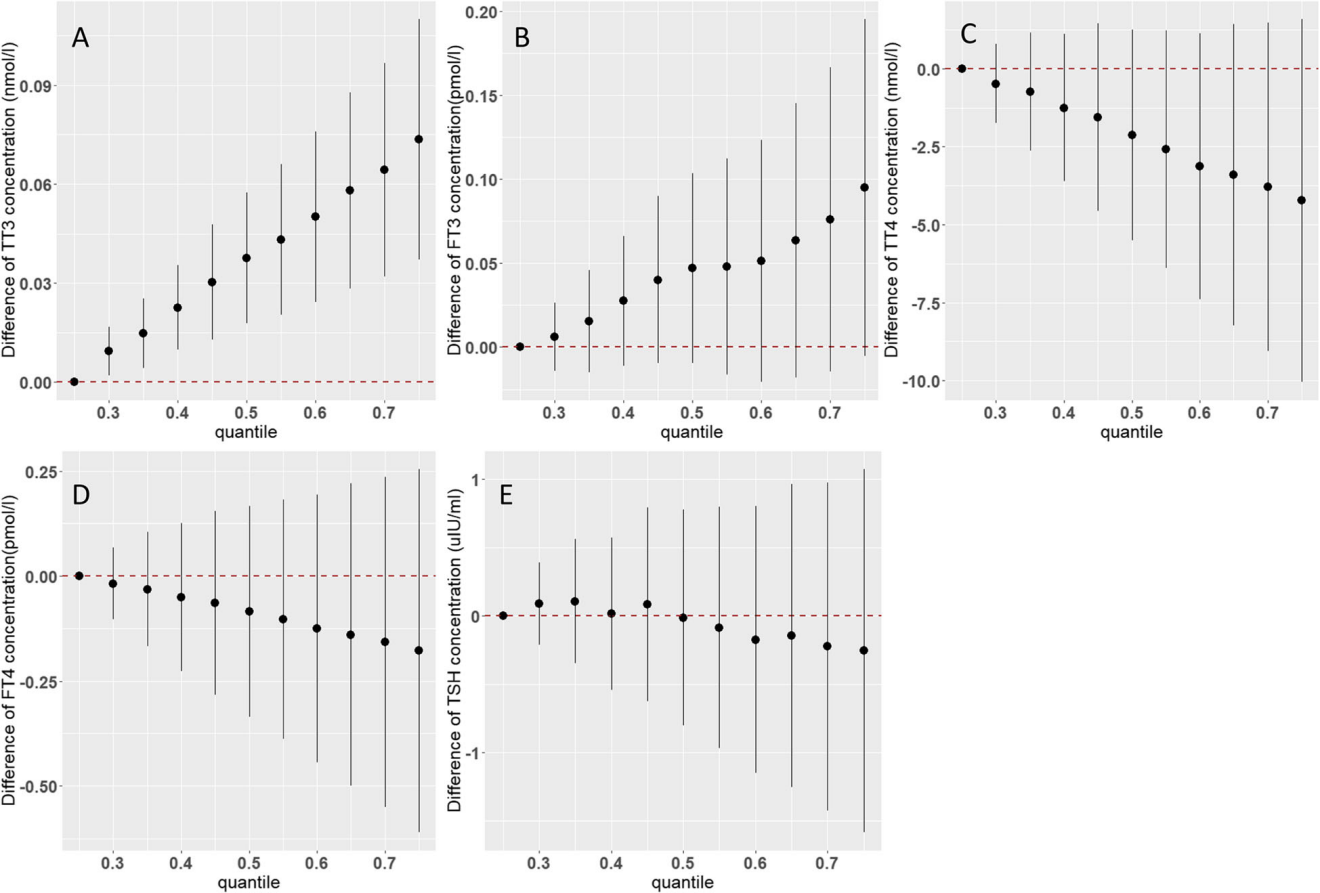
Overall effects of the mixture of eight PFASs on thyroid hormone concentrations in cord blood in Bayesian kernel machine regression models. **a**: T3; **b**: FT3; **c**: T4; **d**: FT4; **e**: TSH. The figure plots the estimated difference in hormone concentrations when all PFAS concentrations are fixed at specified quantiles (ranging from 0.25 to 0.75), as compared to when PFAS concentrations are fixed at the 25th percentile. Dots indicate the estimate, and vertical lines indicate the 95% credible intervals (CrI). All models were adjusted for maternal age at delivery, pre-pregnancy BMI, education, parity, gestational age, delivery type, infant sex, maternal passive smoking during pregnancy, maternal folic acid supplement, and paternal drinking during 3 months before pregnancy

The BKMR model could assess the overall effects of the PFAS mixture on THs when all of the compounds are held at specified quantiles. As indicated in Fig. 2a, higher PFAS mixture concentrations were associated with increased T3 concentrations, such as that compared with the 25th percentile, the 75th percentile of the PFAS mixture was associated with a 0.074 (95% CrI: 0.037, 0.146) nmol/L increase in T3 concentrations. There were suggestive associations between the PFAS mixture and increased FT3 concentrations (estimate at the 75th percentile: 0.095 [95%CrI: − 0.005, 0.195] pmol/L; Fig. 2b). There was a pattern of decreased T4 and FT4 concentrations associated with the PFAS mixture, however, with all CrIs across null (Fig. 2c and d). No association was observed between the PFAS mixture and TSH (Fig. 2e).

**Fig. 3:**
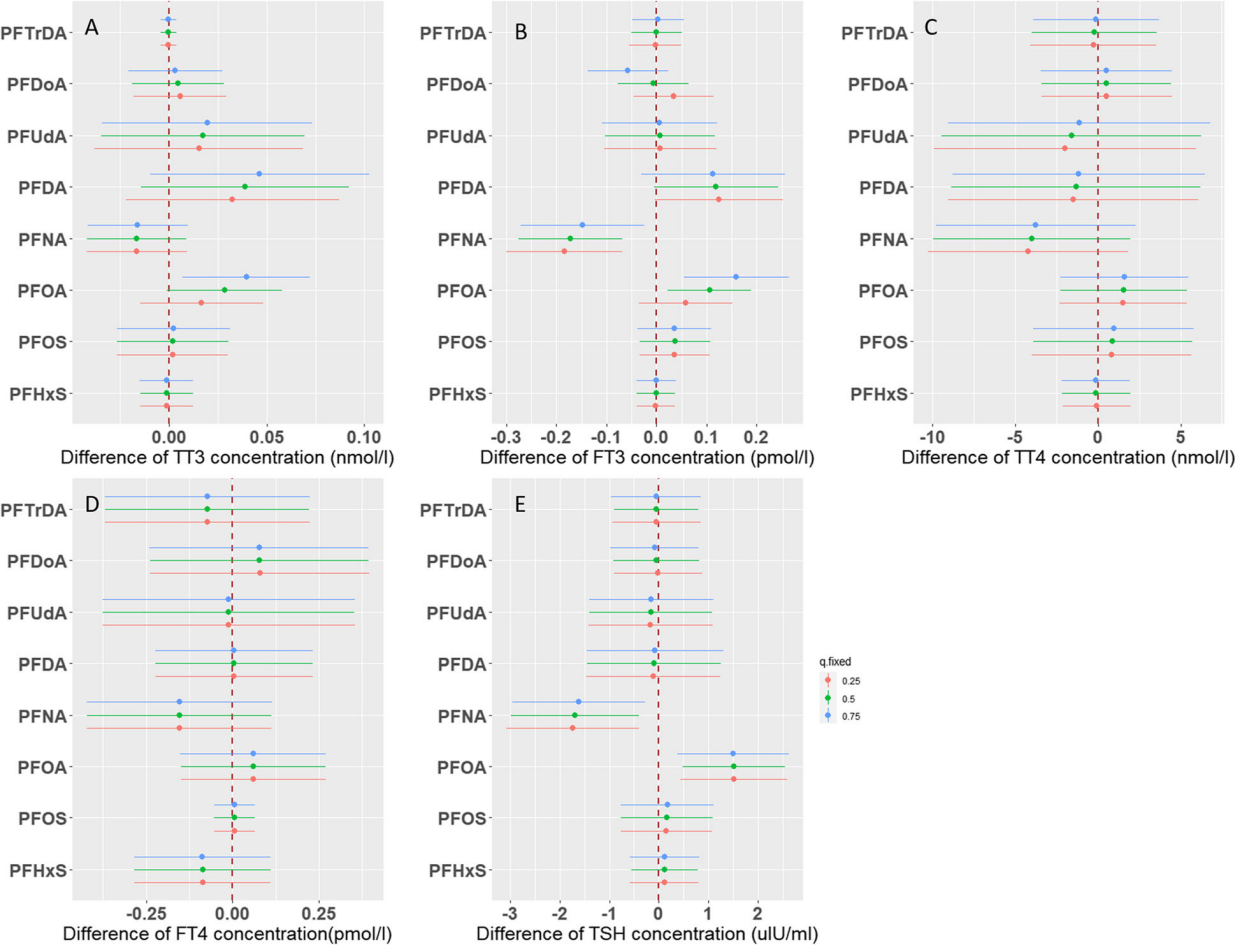
Single-exposure effects of each individual PFAS on thyroid hormone concentrations in cord blood in Bayesian kernel machine regression models. **a**: T3; **b**: FT3; **c**: T4; **d**: FT4; **e**: TSH. This plot describes the estimated difference in hormone concentrations associated with a change in each individual PFAS from its 25th to 75th percentile, when all the other PFASs are fixed at either the 25th (red line), 50th (green line), or 75th percentile (blue line). Dots indicate the estimate, and horizontal lines indicate the 95% credible intervals (CrI). All models were adjusted for maternal age at delivery, pre-pregnancy BMI, education, parity, gestational age, delivery type, infant sex, maternal passive smoking during pregnancy, maternal folic acid supplement, and paternal drinking during 3 months before pregnancy

Figure 3 presents the single-exposure effects of individual PFASs on THs when holding the other compounds at the 25th, 50th, and 75th percentiles, respectively. In PFASs-T3 associations, PFDA had the highest PIP (0.6868), followed by PFUdA (0.3142) and PFOA (0.2116), respectively. When holding all other PFASs at the median concentrations, a change in PFDA, PFUdA and PFOA concentrations from the 25th to 75th percentile was associated with a 0.04 (95%CrI: − 0.01, 0.09), 0.02 (95%CrI: − 0.03, 0.07), and 0.03 (95%CrI: − 0.001, 0.06) nmol/L increase in T3, respectively, although without statistical significance (Fig. 3a). In PFASs-FT3 associations, PFOA, PFNA, and PFDA were the predominant compounds with PIPs of 0.94, 0.781, and 0.7448, respectively. The corresponding FT3 concentration changes associated with a change in PFOA, PFNA and PFDA concentrations from the 25th to 75th percentile were 0.11 (95%CrI: 0.02, 0.19), − 0.17 (95%CrI: − 0.28, − 0.07), and 0.12 (95%CrI: − 0.004, 0.24) pmol/L, respectively (Fig. 3b). Although no individual compound showed PIP > 0.5 in PFASs-TSH associations, PFNA showed the highest PIP (0.4072), followed by PFOA (0.2922). A change in PFNA and PFOA concentrations from the 25th to 75th percentile was associated with a − 1.69 (95%CrI: − 2.98, − 0.41) μIU/mL decrease and a 1.51 (95%CrI: 0.48, 2.55) μIU/mL increase in TSH (Fig. 3e). BKMR analyses did not identify a single, large contributor to the effects on T4 (all PIPs < 0.2) and FT4 concentrations (all PIPs < 0.04; Fig. 3c and d). The effect estimates of PFOA on T3 and FT3 showed shifts in magnitude in the same direction when the other compounds were held at varied quantiles (0.25, 0.50, and 0.75), and those of PFDoA on FT3 showed changes in direction. We further calculated the interactive effect (Supplemental Figure S3), which showed that only the interaction of PFDoA with other PFASs was statistically significant. Most of the associations with T3, FT3, and TSH in BKMR models were similar to those observed in linear regression models. However, BKMR analyses showed that there were associations between PFNA and decreased FT3, and between PFOA and increased TSH, which was not observed in linear regression models. In contrast, some associations shown in linear regression models did not exist in BKMR analyses. Specifically, PFOS, PFNA, and PFDoA contributed much less to the associations with T3 (PIPs ≤0.025). Similarly, the associations between PFOS and FT3 (PIP: 0.037), between PFNA/PFDA/PFUdA and T4 (PIPs < 0.2), and between PFDA/PFUdA and TSH (PIPs < 0.12) attenuated significantly in BKMR models.

**Fig. 4:**
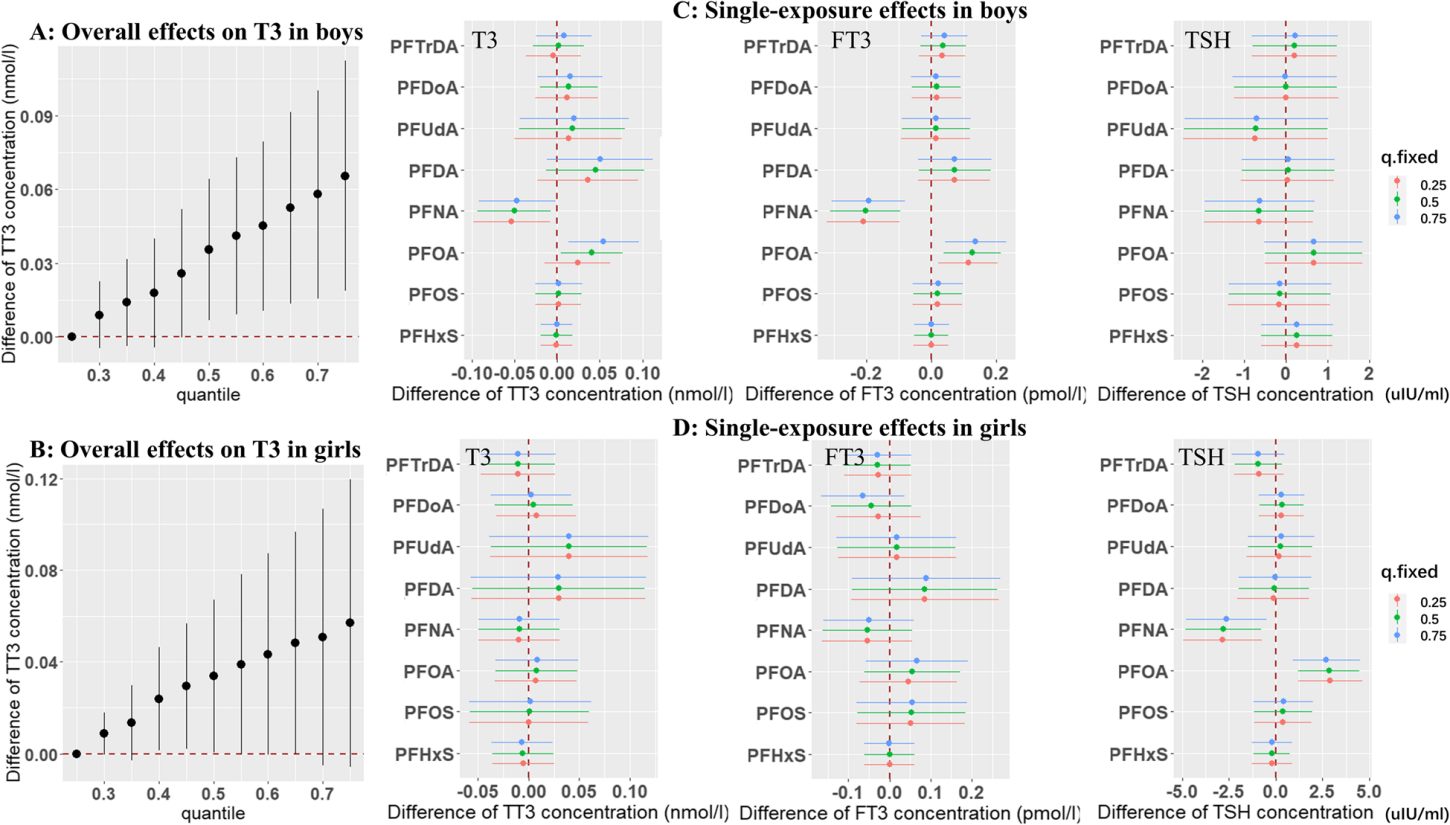
Overall and single-exposure effects of eight PFASs on thyroid hormone concentrations in cord blood in Bayesian kernel machine regression models stratified by infant sex. **a**: Overall effects on T3 in boys; **b**: Overall effects on T3 in girls; **c** Single-exposure effects of each PFAS on T3, FT3, and TSH in boys; **d**: Single-exposure effects of each PFAS on T3, FT3, and TSH in girls. Sub-figure A and B plot the estimated change in T3 concentrations when all PFAS concentrations are fixed at specified quantiles (ranging from 0.25 to 0.75), as compared to when PFAS concentrations are fixed at the 25th percentile. Dots indicate the estimate, and vertical lines indicate the 95% credible intervals (CrI). Sub-figure C and D describe the change in hormone concentrations associated with a change in each individual PFAS from its 25th to 75th percentile, when all the other PFASs are fixed at either the 25th (red line), 50th (green line), or 75th percentile (blue line). Dots indicate the estimate, and horizontal lines indicate the 95% CrIs. All models were adjusted for maternal age at delivery, pre-pregnancy BMI, education, parity, gestational age, delivery type, maternal passive smoking during pregnancy, maternal folic acid supplement, and paternal drinking during 3 months before pregnancy

Figure 4 and Supplemental Figure S4 and S5 present the overall and single-exposure effects of the PFAS mixture on THs, stratified by infant sex. Overall, similar patterns to the pooled gender analyses were observed in each stratum. However, the statistically significant associations between the PFAS mixture and increased T3 concentrations were observed only in boys (Fig. 4a and b). Associations of PFOA and PFNA with T3/FT3 observed in the pooled gender analyses were more pronounced in boys, whereas their associations with TSH were more pronounced in girls (Fig. 4c and d). When the analyses were stratified by delivery type, patterns similar to the pooled analyses were also observed in each stratum in general (Supplemental Figure S6 and S7). However, the associations between the PFAS mixture and T3 were strengthened in infants born via cesarean section with the CrIs ranging from 0.02 (95%CrI: 0.01, 0.03) nmol/L at the 30th percentile of the PFAS mixture to 0.09 (95%CrI: 0.04, 0.14) nmol/L at the 75th percentile, compared with the 25th percentile. Other estimates in the overall effect analyses were across null (Supplemental Figure S6). For single-exposure effects, the associations of PFDA and PFOA with T3 were more pronounced in infants born via caesarean section, while the associations of PFNA with FT3 and of PFOA and PFNA with TSH were mainly observed in infants born via vaginal delivery (Supplemental Figure S7). After further including BPA and Sum_5_PBDEs as co-exposures in BKMR models, the effects of individual PFASs on THs remained similar in all infants (Supplemental Figure S8). Further adjusting for fish intake during the first trimester in BKMR models did not change the observed associations (data not shown).

## Discussion

In the study, we examined the overall and individual effects of maternal exposure to eight PFASs on TH concentrations in cord plasma when accounting for the correlations between multiple PFASs using BKMR models, and also examined the individual effects of eight PFASs using linear regression. In BKMR models, the PFAS mixture was associated with increased T3 and FT3 concentrations. For individual PFAS-hormone associations, maternal PFOA, PFDA, and PFUdA exposure was associated with increased T3 and FT3 (not for PFUdA) concentrations, and maternal PFNA exposure was associated with decreased TSH concentrations in both BKMR and linear regression models. BKMR models further identified that there were PFNA association with decreased FT3 concentrations and PFOA association with increased TSH concentrations. The associations of PFOA and PFNA with T3/FT3 were more pronounced in boys, while their associations with TSH were more pronounced in girls.

The observed associations from linear regression models of maternal PFOS, PFNA, and PFDoA exposure with increased T3 concentrations, PFOS and PFUdA with increased FT3 concentrations, PFNA, PFDA and PFUdA with decreased T4 concentrations, and PFDA and PFUdA with decreased TSH concentrations were not observed in BKMR models. These associations are possibly spurious because linear regression models could not deal with either high correlations between multiple PFASs or non-linear dose-responses that may bias the associations.

Our findings are not consistent with reports from rodent studies, which tend to report decreased T4 and unchanged T3/TSH in rat pups following prenatal PFAS exposure [13, 14, 16]. However, studies in zebrafish have reported increased T3 and unchanged T4 in the F(0) and F(1) generations following PFAS exposure [31, 32]. Interspecies differences and exposure doses in most animal studies that are significantly higher than those experienced by the human population may make it difficult to compare the effects of PFASs on thyroid function between animals and humans.

Only a few studies have used multi-pollutant models to address the correlations between multiple PFASs when examining their effects on THs in infants. Aimuzi et al’s study [21] used sparse partial least squares regression to identify the dominant PFASs related to FT4, FT3, and TSH in cord blood, while the model did not estimate the joint effects of the PFAS mixture. Two studies from the US [33, 34] also used BKMR to assess the overall effects of prenatal PFAS mixture exposure on neonatal THs. The study based on the Project Viva cohort [33] showed a statistically non-significant inverse association between the PFAS mixture and lower T4 concentrations (T3 and FT3 not measured) in heel stick blood and no association with TSH, which is consistent with our findings. However, the study based on the HOME study [34] did not find any association between the PFAS mixture (PFOA, PFOS, PFNA, and PFHxS) and any TH in cord blood. For the single exposure effects of PFASs, our results were partially consistent with those of previous epidemiological studies. In this study, maternal PFAS exposure was associated with higher FT3 and T3 concentrations, which is consistent with three previous studies where maternal PFDA exposure was associated with higher FT3 among girls born via vaginal delivery [24], and cord blood PFDA and PFUdA were associated with higher T3 concentrations [22, 35]. In addition, our finding that maternal PFNA was associated with decreased cord TSH is consistent with three previous studies [21, 22, 36], and that of maternal PFOA being associated with increased TSH is consistent with Kim’s study [12]. Other studies that have examined PFAS exposure and TH concentrations tended to report that PFAS exposure was associated with decreased T4 (or FT4) and/or T3 with different compounds showing statistical significance [11, 19, 20, 34, 35].

We should bear in mind that it is quite difficult to directly compare our study with previous studies, due to differences in the timing and matrix of PFAS measurements (maternal blood at first, second, or third trimester vs. cord blood), measured PFASs and their concentrations, measured TH types and matrix (type: five THs vs. only T4/TSH measured, T4, T3, and TSH measured; matrix: cord blood vs. neonatal heel stick blood), characteristics of the study population (all infants vs. born via vaginal delivery or pre-labor cesarean section), and models used for analyses. Similar to another study conducted in Shanghai [21] but different from most previous studies, PFOA, but not PFOS, was the predominant compound in our study, and its concentration was much higher (median: 19.4 vs. 2.0–5.6 ng/mL) than previously reported [11, 19, 20, 24]. This may partly explain why associations between PFOA and THs were observed in this study. In addition, TH concentrations measured from cord blood and neonatal heel stick may be different since the stress during birth could cause TSH surge in cord blood. As shown in this study and previous literature [29], infants born via vaginal delivery had statistically significant higher TSH and lower FT3 concentrations than those born via cesarean section. Further stratified analyses by delivery mode showed that the PFAS-T3 associations were more pronounced in infants born via cesarean section, while PFAS-FT3/TSH associations were more pronounced in those born via vaginal delivery. Thus, the methodological difference in TH measurement and the characteristics of the population may lead to the difference in PFAS-TH associations to some extent. In addition, BKMR in the study included eight PFASs simultaneously and allowed the disentanglement of the independent effects of each compound among the co-exposed and highly correlated PFAS mixture. Most previous studies used conventional linear regression in a one-at-a-time approach without considering the high correlations between PFASs. The application of varied models may produce a different result, as shown in this study.

The underlying mechanism by which PFAS exposure disrupts thyroid homeostasis is still not clear. Toxicological studies have suggested several potential mechanisms. PFASs have a competitive binding ability with TH transport protein transthyretin [37], may enhance hepatic uptake and metabolism of T4 via the upregulation of uridine diphosphoglucuronosyl transferase 1A1 and hepatic organic anion transporter [38, 39], and increase the conversion of T4 to T3 via the upregulation of type 1 deiodinase [38]. These mechanisms may contribute to increased T3 and lower T4 concentrations following maternal PFAS exposure. However, the mechanisms could not explain all the findings since PFNA and PFOA were associated with T3, FT3, and TSH in opposite directions. Furthermore, most of the toxicological studies were based on PFOS exposure, while our study did not find associations between PFOS and THs. Some toxicological studies have suggested that individual PFASs may have different potency and mechanisms for exerting their disrupting effects, and their effects on THs need to be further clarified.

BKMR models suggested sex-specific effects of PFASs on THs, manifesting with more pronounced associations of PFOA and PFNA with T3/FT3 in boys and with TSH in girls. Sex-specific effects have also been observed in previous studies [21, 22]. Sexual hormones may influence TH concentrations by increasing serum thyroxine-binding globulin concentrations [40]. PFASs can affect estrogen and androgen receptor transactivity [41], interfere with steroidogenesis, and inhibit testosterone release from Leydig cells [42], leading to changes in estradiol (E2) and testosterone (T) concentrations. In humans, prenatal PFAS exposure is associated with decreased cord T/E2, progesterone, and inhibin B, and with increased E2 concentrations sex-specifically [43]. We speculated that affected sexual hormones might further influence thyroid homeostasis sex-specifically. However, the exact underlying mechanism has not yet been elucidated.

The major strength of the study was the prospective design that could provide potential causality of the associations between prenatal PFAS exposure and fetal THs. In addition, we applied multi-pollutant models of a BKMR approach to estimate the overall, single-exposure, and interactive effects of multiple PFASs on THs, addressing the high correlation of the PFAS mixture and the potential non-linear dose-responses.

The findings should also be interpreted in light of certain limitations. First, the study included a limited number of subjects (24%) from the S-MBCS cohort, mainly because quite a few cord blood samples were not collected due to practical reasons, and samples were further selected for TH measurement due to limited funding. It is less likely that these reasons for attrition are associated with both maternal PFAS concentrations and cord blood TH concentrations. Furthermore, compared with those excluded, the included mother-infant pairs had similar characteristics except for a slightly higher proportion of preterm births, and similar PFAS distributions. The attrition seemed to be non-differential, which alleviated our concern regarding selection bias. Second, we measured TH concentrations in cord blood rather than neonatal blood. A neonatal TSH surge occurs at delivery caused by intrapartum fetal stress, and neonates born vaginally had higher TSH and lower FT3 concentrations in cord blood than those born via cesarean section. Therefore, we stratified the analyses by delivery type to reduce the influence due to TSH surge, especially during vaginal delivery. Third, we could not control potential confounding effects of other unmeasured EDCs co-exposed with PFASs, if they are correlated with PFASs. In addition, we did not measure maternal iodine status, which is an important determinant of neonatal thyroid function. However, we further adjusted for fish intake (one kind of iodine-rich food) in BKMR models, and the results remained similar. At last, although we measured the concentrations of five THs, we did not measure cord blood concentrations of thyroid transport proteins and thyroid antibodies; thus, we could not address the overall picture of associations related to fetal thyroid hormone homeostasis.

## Conclusion

In this study, prenatal exposure to the PFAS mixture was associated with increased T3 and FT3 concentrations in cord blood. In terms of the predominant compounds, maternal PFOA, PFDA, and PFUdA exposure was associated with increased T3 and FT3 (not for PFUdA), accompanied by PFOA associated with increased TSH. In contrast, PFNA was associated with decreased FT3 and TSH concentrations. The effects of PFOA and PFNA on THs may be sex-specific, with more pronounced effects on FT3/T3 in boys and TSH in girls. These findings suggest that prenatal co-exposure to multiple PFASs may affect fetal thyroid hormones, and individual PFASs have varied effects—differing in magnitude and direction—on fetal thyroid hormones.

## Supplementary Information

**Additional file 1:** **Figure S1** Adjusted generalized additive model plots of ln-transformed PFAS concentrations with thyroid hormone concentrations. **Figure S2** The causal network between maternal plasma PFAS concentrations and thyroid hormones (TH) in cord plasma, presented in a directed acyclic graph. **Figure S3** Interactive effects of each PFAS with other remaining compounds in the associations of maternal PFAS concentrations with total triiodothyronine (T3; A) and free T3 (FT3; B) concentrations. **Figure S4** Overall effects of the mixture of eight PFASs on thyroid hormone concentrations in cord blood in Bayesian kernel machine regression models stratified by infant sex. **Figure S5** Single-exposure effects of each individual PFAS on thyroid hormone concentrations in cord blood in Bayesian kernel machine regression models stratified by infant sex. **Figure S6** Overall effects of the mixture of eight PFASs on thyroid hormone concentrations in cord blood in Bayesian kernel machine regression models stratified by type of delivery. **Figure S7** Single-exposure effects of each individual PFAS on thyroid hormone concentrations in cord blood in Bayesian kernel machine regression models stratified by type of delivery. **Figure S8** Single-exposure effects of each individual PFAS, bisphenol A (BPA), and polybrominated diphenyl ethers (PBDEs) on thyroid hormone concentrations in cord blood in Bayesian kernel machine regression models. **Table S1** Comparisons of maternal plasma PFAS concentrations between mother-infant pairs included and excluded from the present study using Mann–Whitney U test. **Table S2** Pearson correlation coefficients between pairs of ln-transformed PFAS concentrations (ng/mL) in maternal plasma. **Table S3** Pearson correlation coefficients between pairs of thyroid hormones in 300 cord plasma samples. **Table S4** Thyroid hormone concentrations in cord plasma among infants born via vaginal delivery or caesarean section. **Table S5** Associations between maternal plasma PFAS concentrations (tertile, ng/mL) and thyroid hormones in cord plasma by linear regression models (*n* = 280).

## Data Availability

The datasets used in the current study are available from the corresponding authors on reasonable request.

## Abbreviations

BKMR: Bayesian kernel machine regression
BMI: Body mass index (kg/m2)
CI: Confidence interval
CrI: Credible Interval
FT3: Free triiodothyronine
FT4: Free thyroxine
GAM: Generalized additive model
GM: Geometric means
LODs: Limits of detection
PFASs: Perfluoroalkyl substances
PFDA: Perfluorodecanoic acid
PFDoA: Perfluorododecanoic acid
PFHxS: Perfluorohexane sulfonate
PFNA: Perfluorononanoic acid
PFOA: Perfluorooctanoic acid
PFOS: Perfluorooctane sulfonate
PFTrDA: Perfluorotridecanoic acid
PFUdA: Perfluoroundecanoic acid
PIPs: Posterior inclusion probabilities
S-MBCS: Shanghai-Minhang Birth Cohort Study
T3: Total triiodothyronine
T4: Total thyroxine
THs: Thyroid hormones
TSH: Thyroid stimulating hormone
