# Supplementary material for: Prenatal exposure to perfluoroalkyl substances and thyroid hormone concentrations in cord plasma in a Chinese birth cohort
Source: Environ Health. 2020 Nov 26;19:127. doi: 10.1186/s12940-020-00679-7 (PMC7690128; doi:10.1186/s12940-020-00679-7)
Supplement: Supplementary file 1 — Additional file 1: Figure S1 Adjusted generalized additive model plots of ln-transformed PFAS concentrations with thyroid hormone concentrations. Figure S2 The causal network between maternal plasma PFAS concentrations and thyroid hormones (TH) in cord plasma, presented in a directed acyclic graph. Figure S3 Interactive effects of each PFAS with other remaining compounds in the associations of maternal PFAS concentrations with total triiodothyronine (T3; A) and free T3 (FT3; B) concentrations. Figure S4 Overall effects of the mixture of eight PFASs on thyroid hormone concentrations in cord blood in Bayesian kernel machine regression models stratified by infant sex. Figure S5 Single-exposure effects of each individual PFAS on thyroid hormone concentrations in cord blood in Bayesian kernel machine regression models stratified by infant sex. Figure S6 Overall effects of the mixture of eight PFASs on thyroid hormone concentrations in cord blood in Bayesian kernel machine regression models stratified by type of delivery. Figure S7 Single-exposure effects of each individual PFAS on thyroid hormone concentrations in cord blood in Bayesian kernel machine regression models stratified by type of delivery. Figure S8 Single-exposure effects of each individual PFAS, bisphenol A (BPA), and polybrominated diphenyl ethers (PBDEs) on thyroid hormone concentrations in cord blood in Bayesian kernel machine regression models. Table S1 Comparisons of maternal plasma PFAS concentrations between mother-infant pairs included and excluded from the present study using Mann–Whitney U test. Table S2 Pearson correlation coefficients between pairs of ln-transformed PFAS concentrations (ng/mL) in maternal plasma. Table S3 Pearson correlation coefficients between pairs of thyroid hormones in 300 cord plasma samples. Table S4 Thyroid hormone concentrations in cord plasma among infants born via vaginal delivery or caesarean section. Table S5 Associations between maternal plasma PFAS concentrations ( [file 12940_2020_679_MOESM1_ESM.docx]

**Prenatal exposure to perfluoroalkyl substance and thyroid hormone concentrations in cord plasma in a Chinese birth cohort**

Hong Liang^1^, Ziliang Wang^1^, Maohua Miao^1^, Youping Tian^2^, Yan Zhou^3^, Sheng Wen^3^, Yao Chen^1^, Xiaowei Sun^1^, Wei Yuan^1^*

**Supplementary materials**

**Figure S1** Adjusted generalized additive model plots of ln-transformed PFAS concentrations with thyroid hormone concentrations.

**Figure S2** The causal network between maternal plasma PFAS concentrations and thyroid hormones (TH) in cord plasma, presented in a directed acyclic graph.

**Figure S3** Interactive effects of each PFAS with other remaining compounds in the associations of maternal PFAS concentrations with total triiodothyronine (T3; A) and free T3 (FT3; B) concentrations.

**Figure S4** Overall effects of the mixture of eight PFASs on thyroid hormones concentrations in cord blood in Bayesian kernel machine regression models stratified by infant sex.

**Figure S5** Single-exposure effects of each individual PFAS on thyroid hormone concentrations in cord blood in Bayesian kernel machine regression models stratified by infant sex.

**Figure S6** Overall effects of the mixture of eight PFASs on thyroid hormone concentrations in cord blood in Bayesian kernel machine regression models stratified by type of delivery.

**Figure S7** Single-exposure effects of each individual PFAS on thyroid hormone concentrations in cord blood in Bayesian kernel machine regression models stratified by type of delivery.

**Figure S8** Single-exposure effects of each individual PFAS, bisphenol A (BPA), and bolybrominated diphenyl ethers (Sum_5_PBDEs) on thyroid hormone concentrations in cord blood in Bayesian kernel machine regression models.

**Table S1** Comparisons of maternal plasma PFAS concentrations between mother-infant pairs included and excluded from the present study using Mann–Whitney U test.

**Table S2** Pearson correlation coeﬃcients between pairs of ln-transformed PFAS concentrations (ng/mL) in maternal plasma.

**Table S3** Pearson correlation coeﬃcients between pairs of thyroid hormones in 300 cord plasma samples.

**Table S4** Thyroid hormone concentrations in cord blood among infants born via vaginal delivery or caesarean section.

**Table S5** Associations between maternal plasma PFAS concentrations (tertile, ng/mL) and thyroid hormones in cord plasma by linear regression models (n=280).


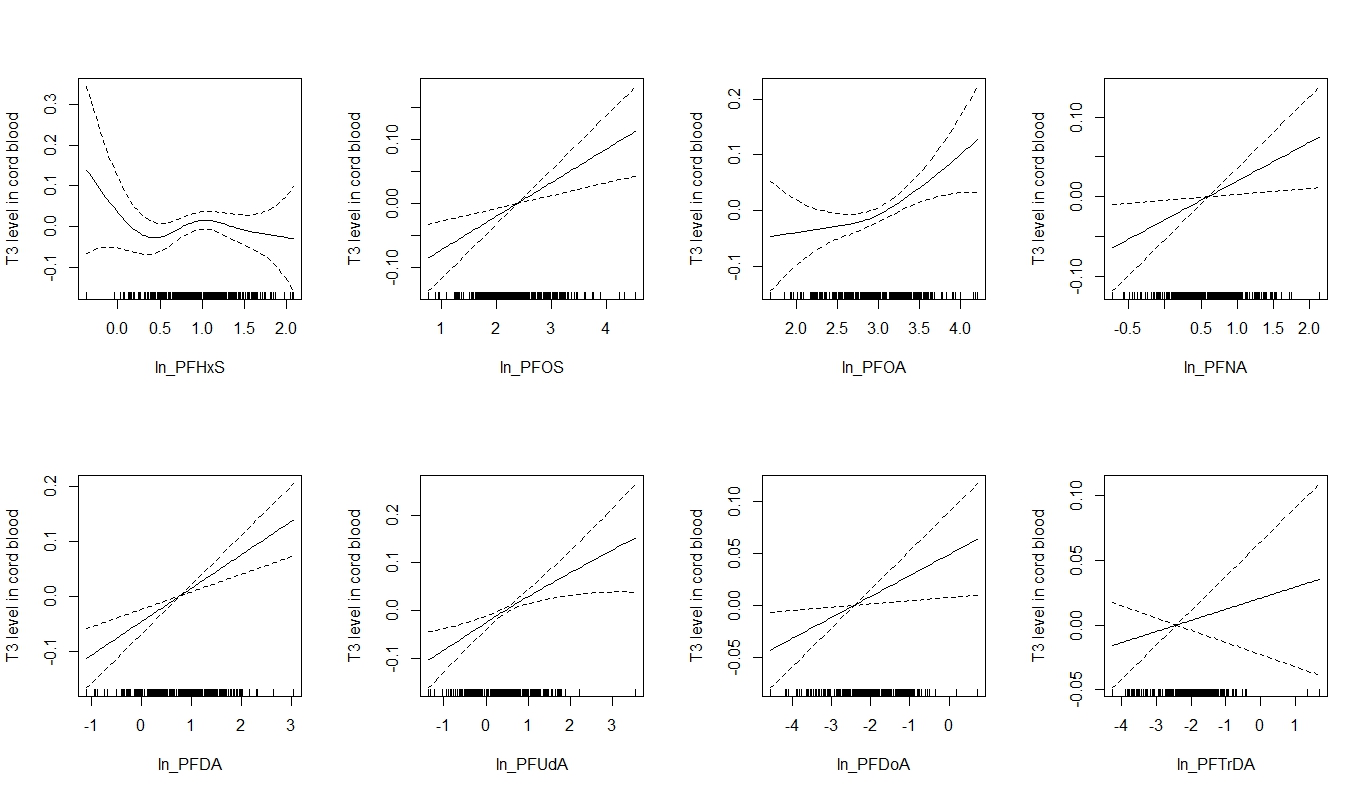

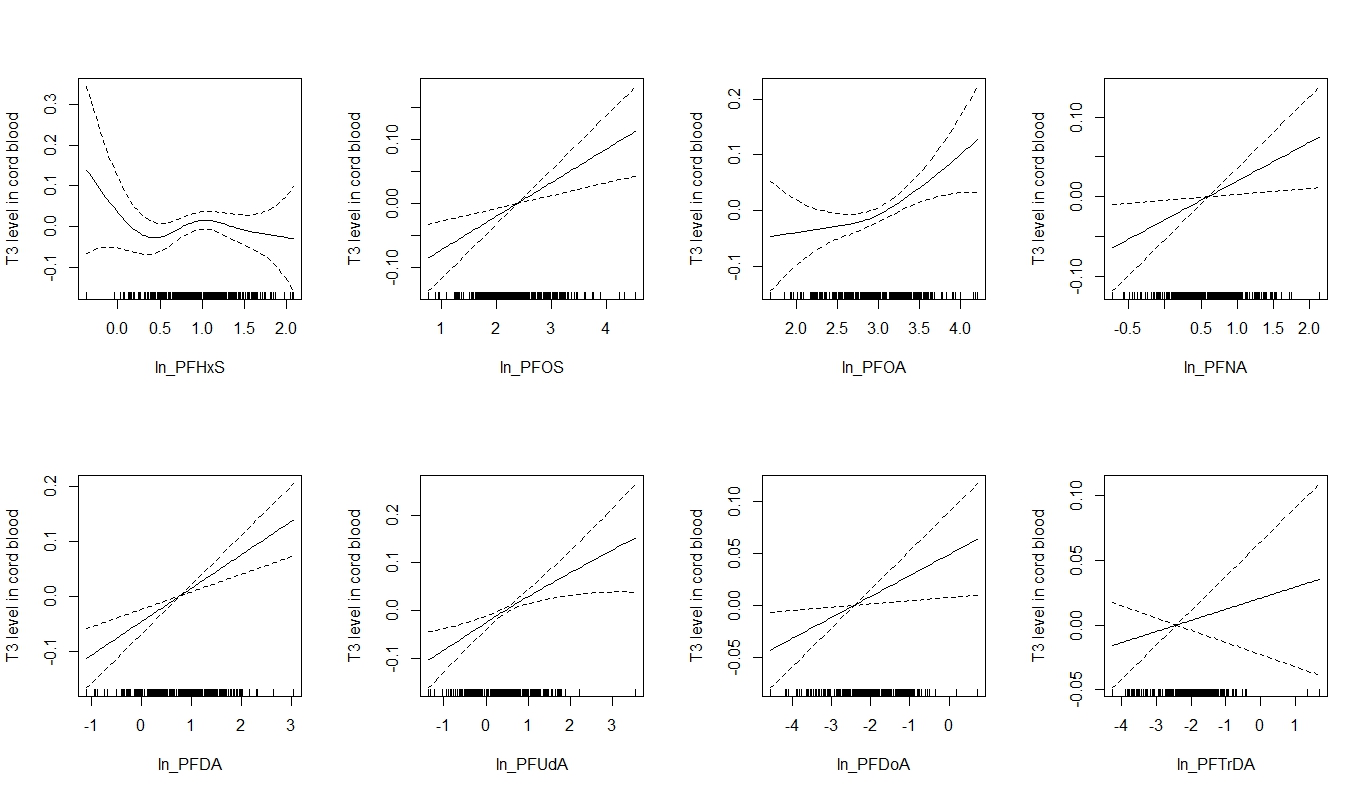

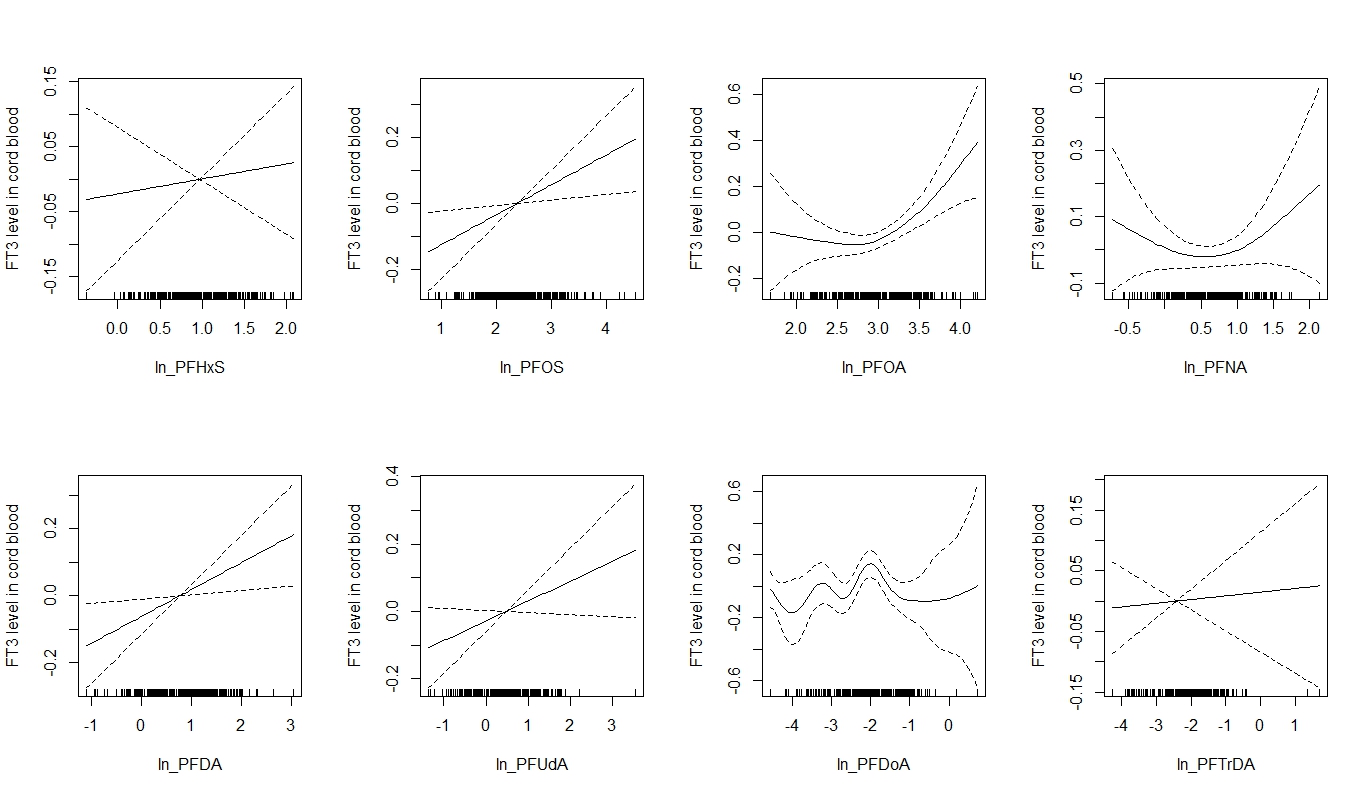

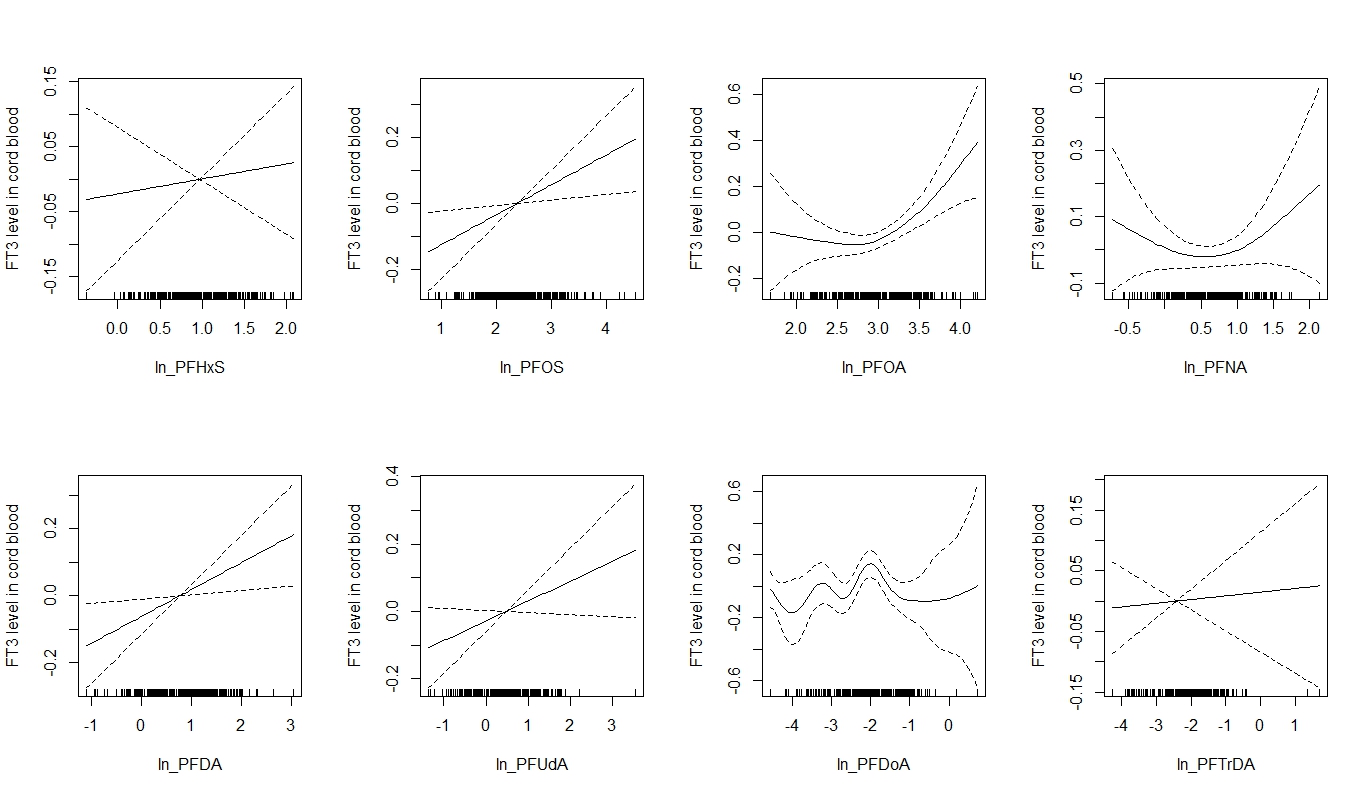

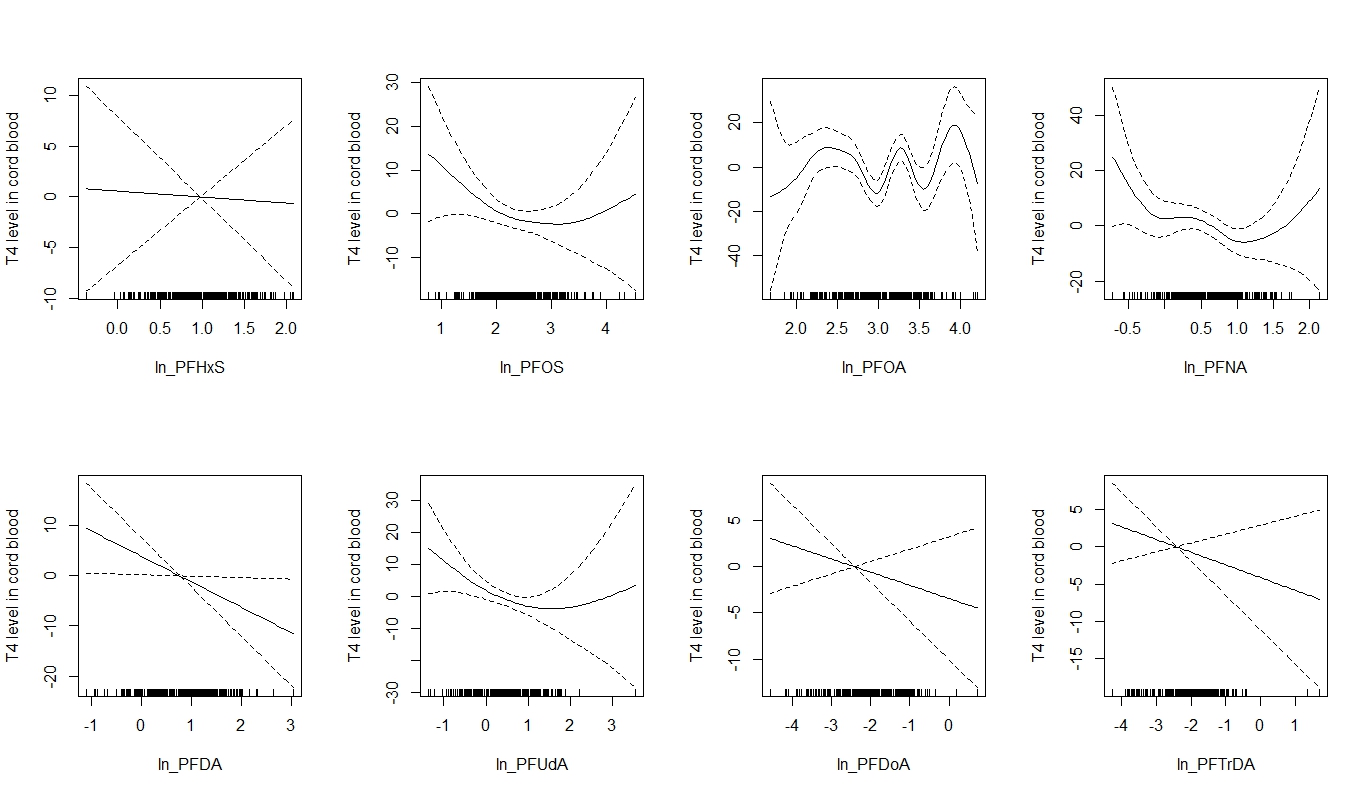

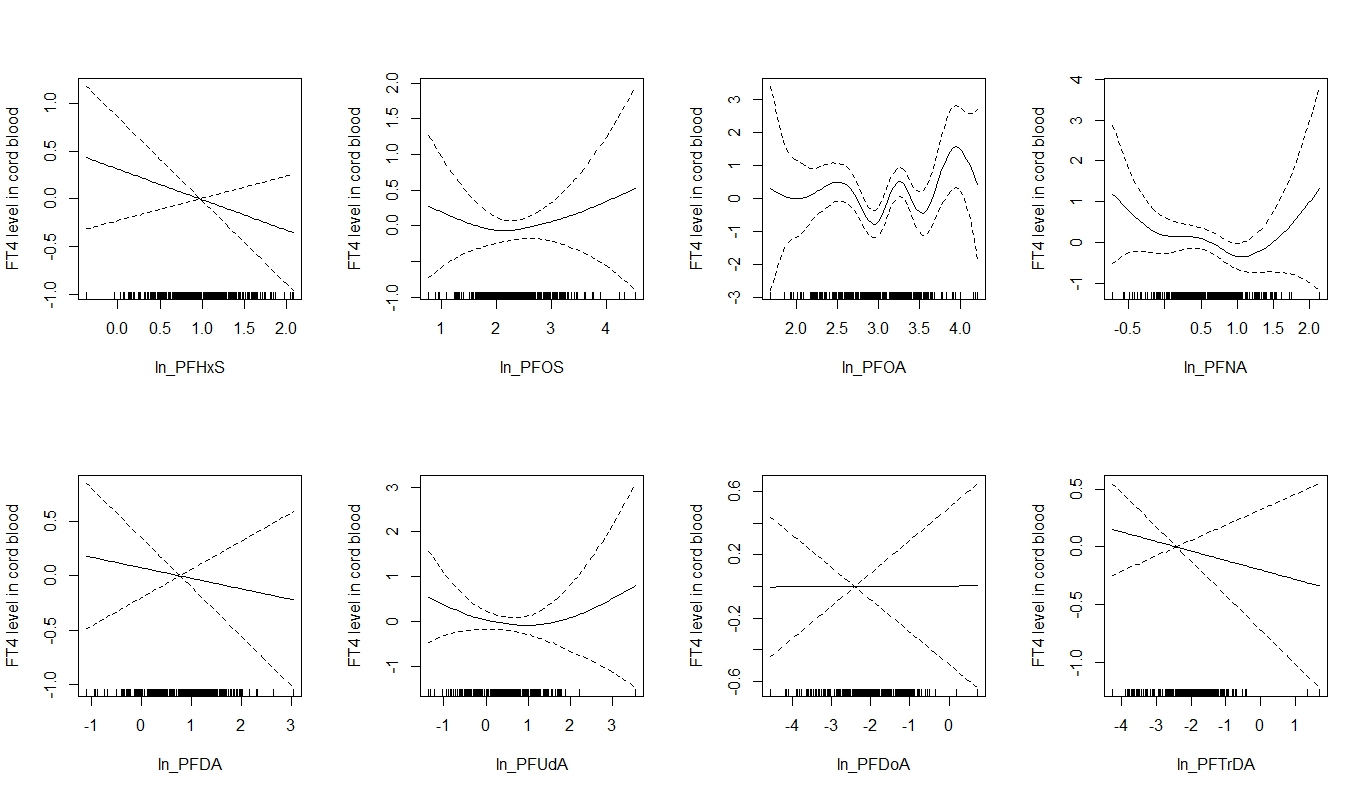

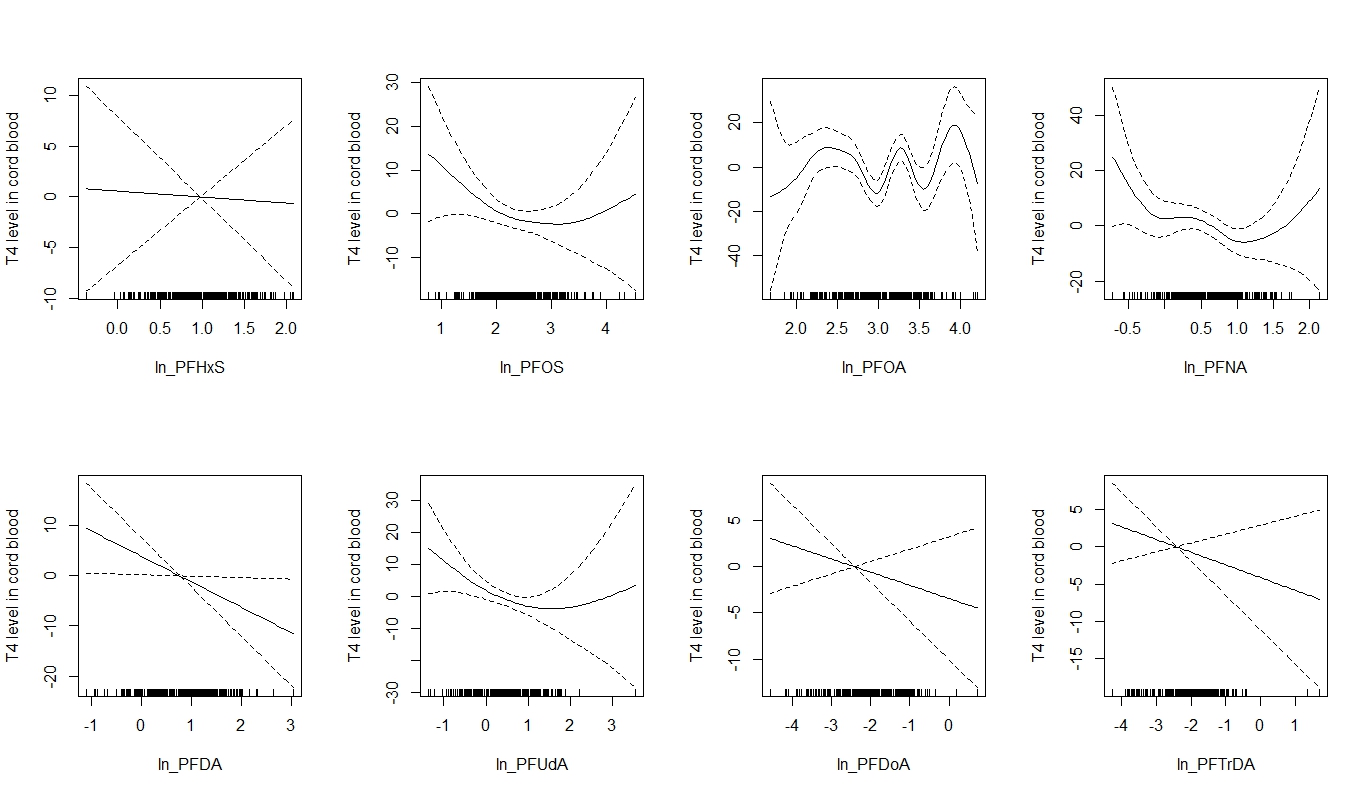

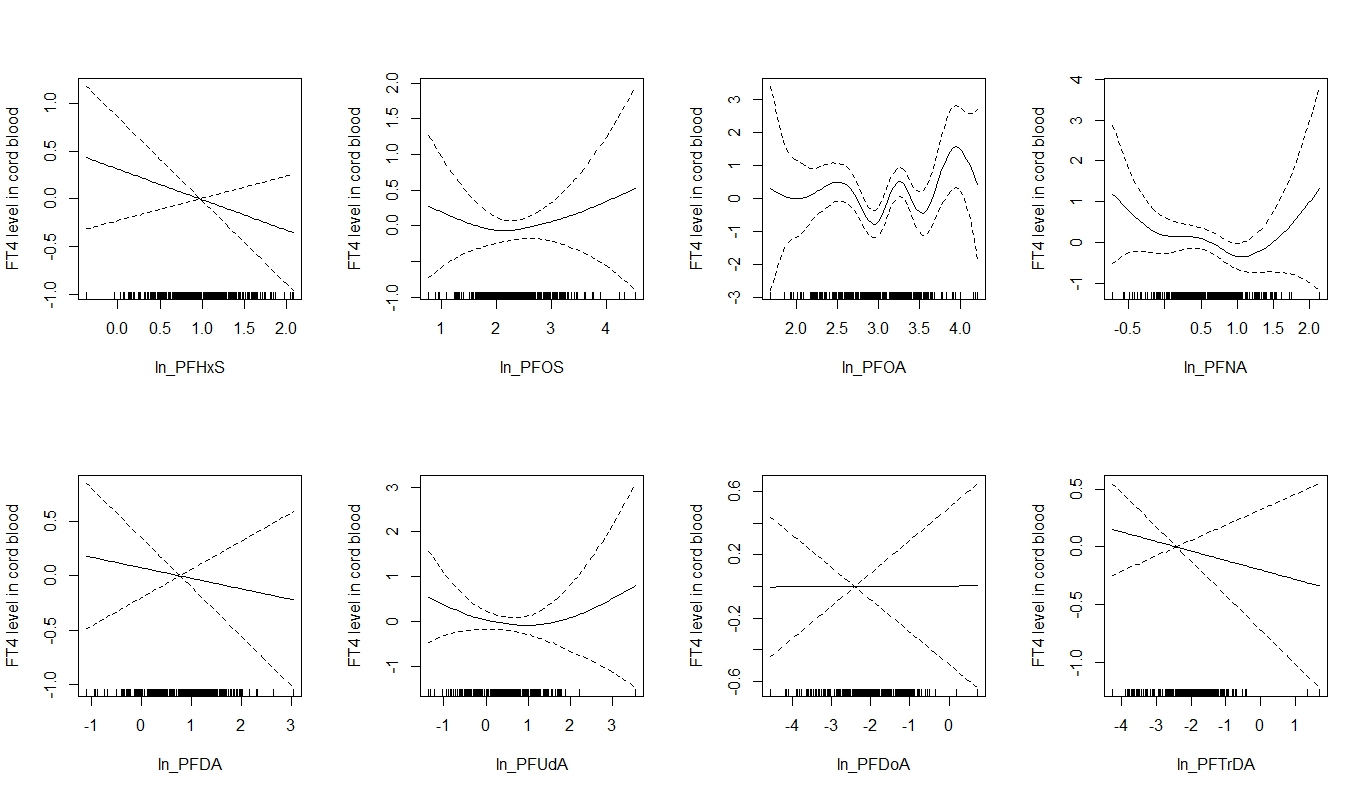

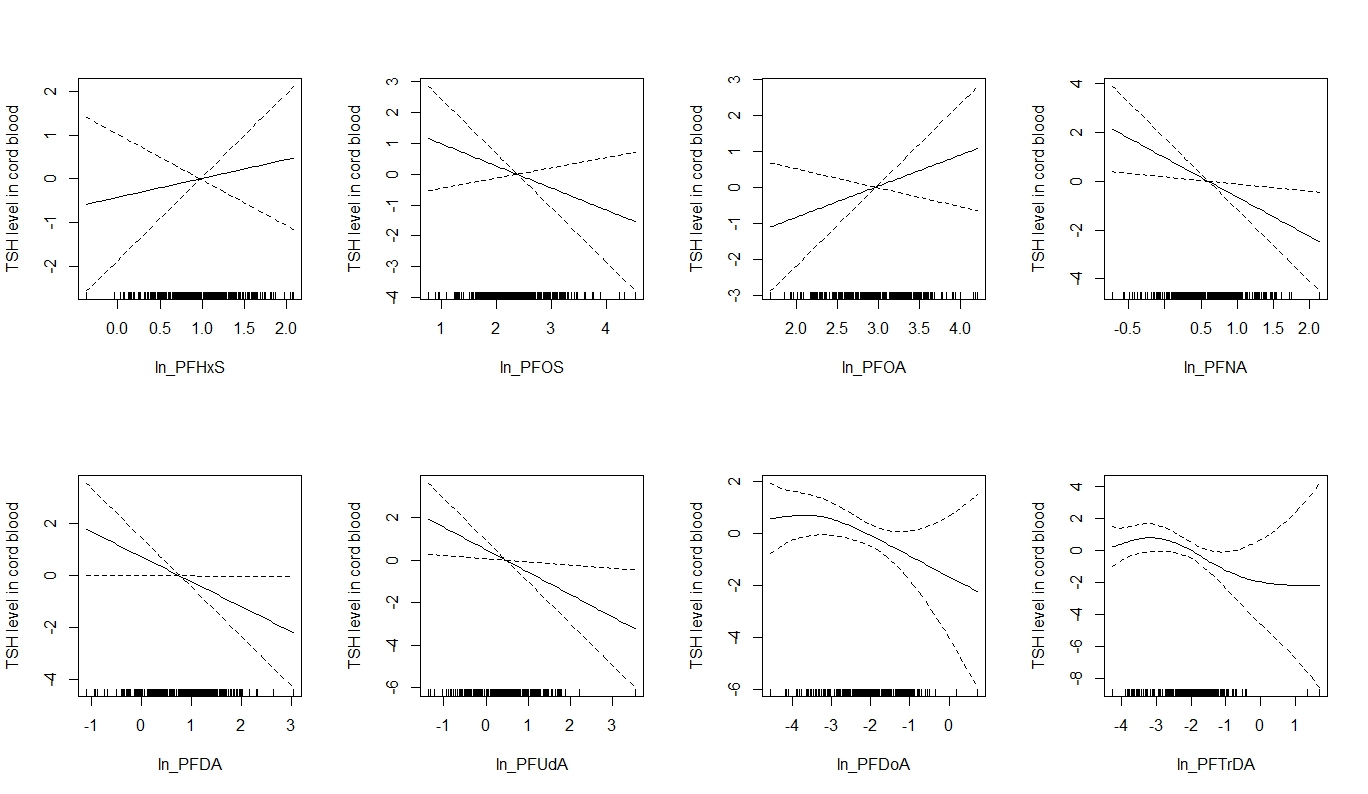


**Figure S1** Adjusted generalized additive model plots of ln-transformed PFAS concentrations with thyroid hormone concentrations. The middle line in each panel shows the estimated effect (y-axis) of maternal PFAS concentrations on hormone concentrations at each time point plotted against the ln-transformed PFAS concentrations (x-axis), after adjusting for covariates. Upper and lower lines show 95% CI of the estimated effect. T3, total triiodothyronine; T4, total thyroxine; FT3, free triiodothyronine; FT4, free thyroxin; TSH, thyroid stimulating hormone.


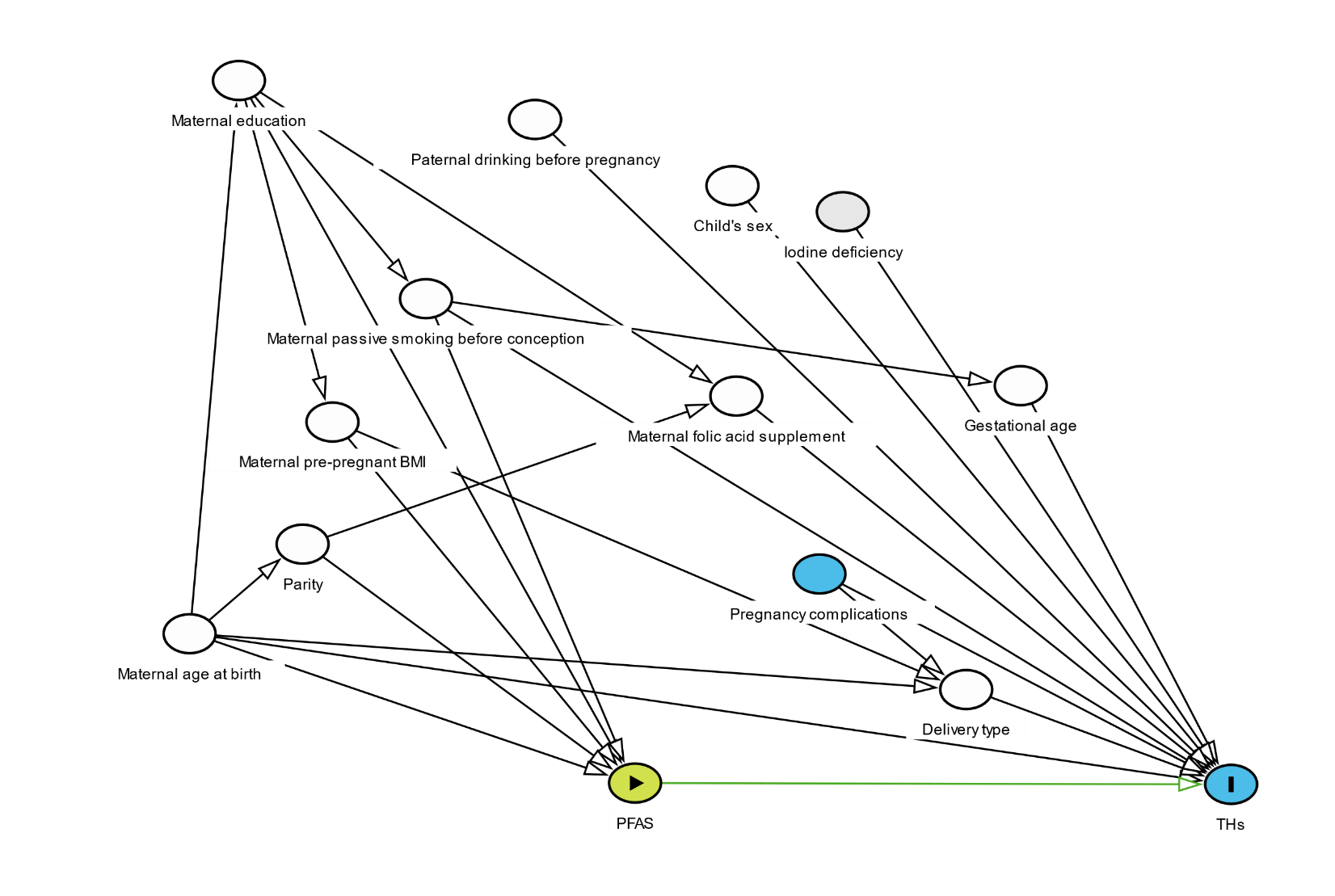
**Figure S2** The causal network between maternal plasma PFAS concentrations and thyroid hormones (TH) in cord plasma, presented in a directed acyclic graph. Adjusted for maternal age at birth, maternal pre-pregnancy BMI, maternal education, parity, maternal passive smoking before conception, maternal folic acid supplement, paternal drinking before pregnancy, gestational age, delivery type, and infant sex.


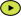
exposure,
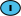
 outcome,
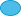
 ancestor of outcome,
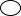
 adjusted variable,
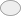
 unobserved variable,
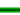
causal path.

B

A


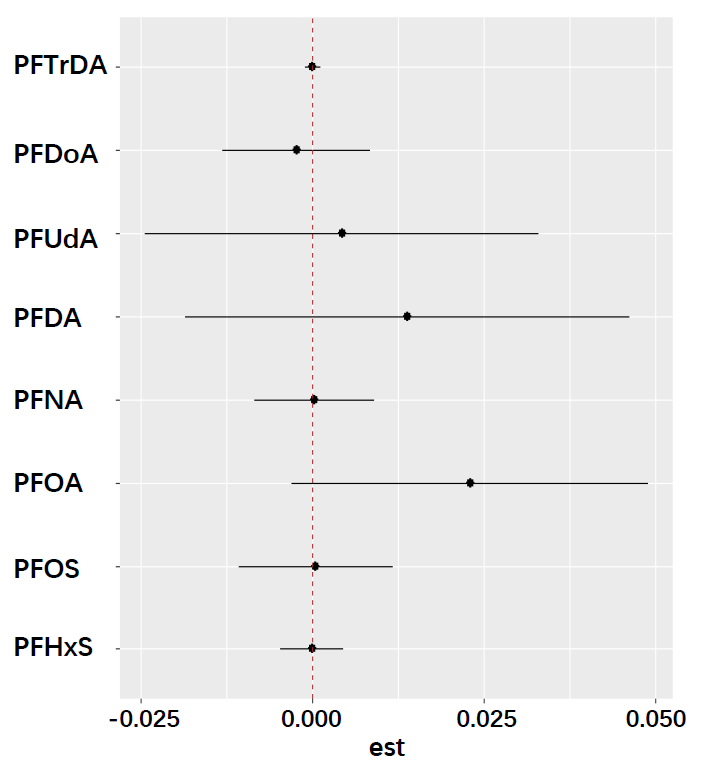

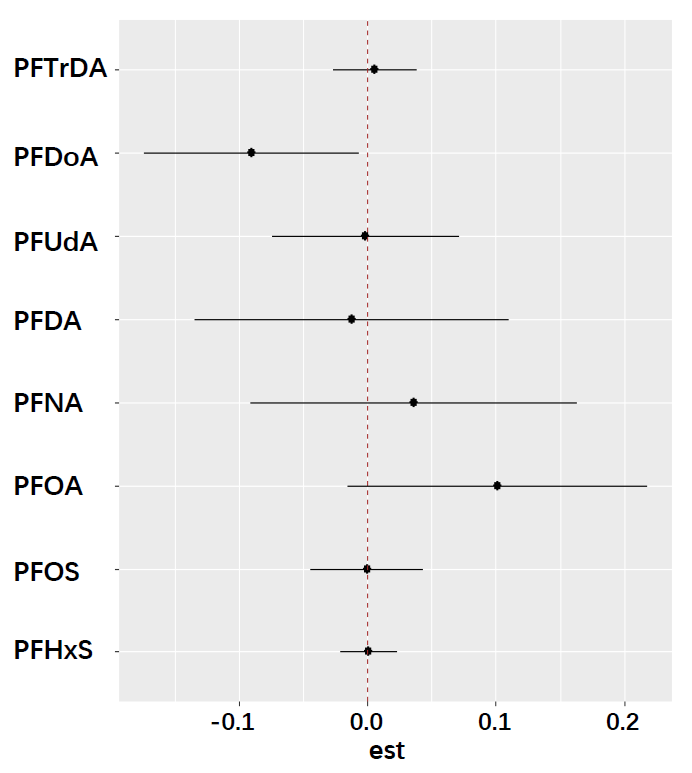


**Figure S3** Interactive effects of each PFAS with other remaining compounds in the associations of maternal PFAS concentrations with total triiodothyronine (T3; A) and free T3 (FT3:B) concentrations.

Note: Interactive effect is defined as the change in the single-exposure health effects when all of the remaining PFAS concentrations are fixed at their 25th percentile as compared to when they are fixed at their 75^th^ percentile. Dots indicate the estimate, and horizontal lines indicate the 95% credible intervals (CrI).

All models were adjusted for maternal age at delivery, pre-pregnancy BMI, education, parity, gestational age, delivery type, infant sex, maternal passive smoking during pregnancy, maternal folic acid supplement, and paternal drinking during three months before pregnancy.

**Figure S4.** Overall effects of the mixture of eight PFASs on thyroid hormones concentrations in cord blood in Bayesian kernel machine regression models stratified by infant sex (A: in boys; B: in girls).

TSH

FT4

T4

TSH

FT4

FT3

T4

FT3


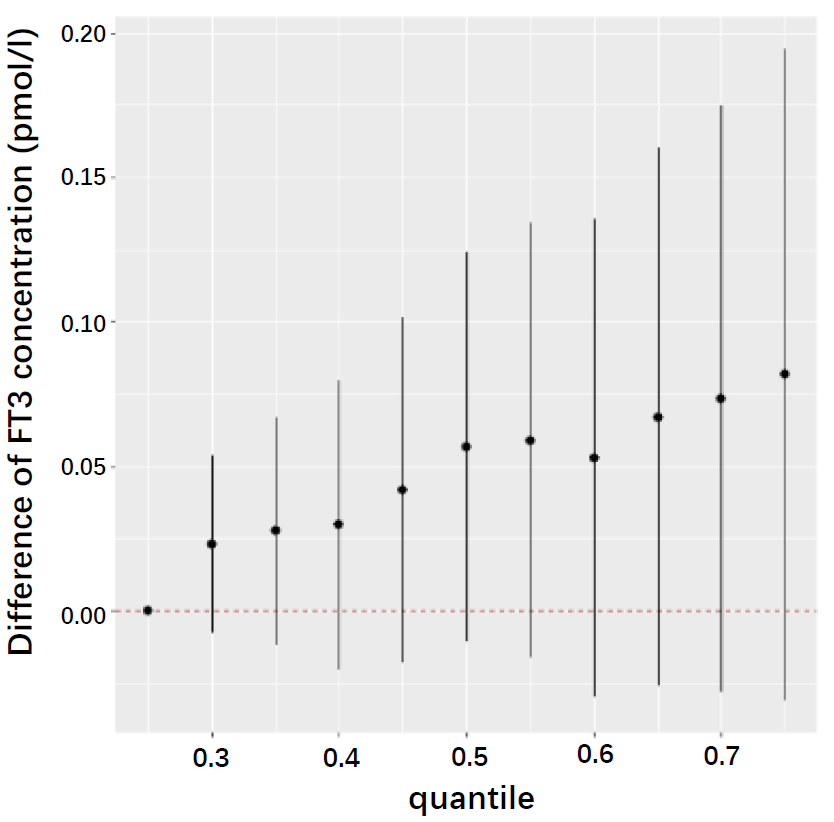

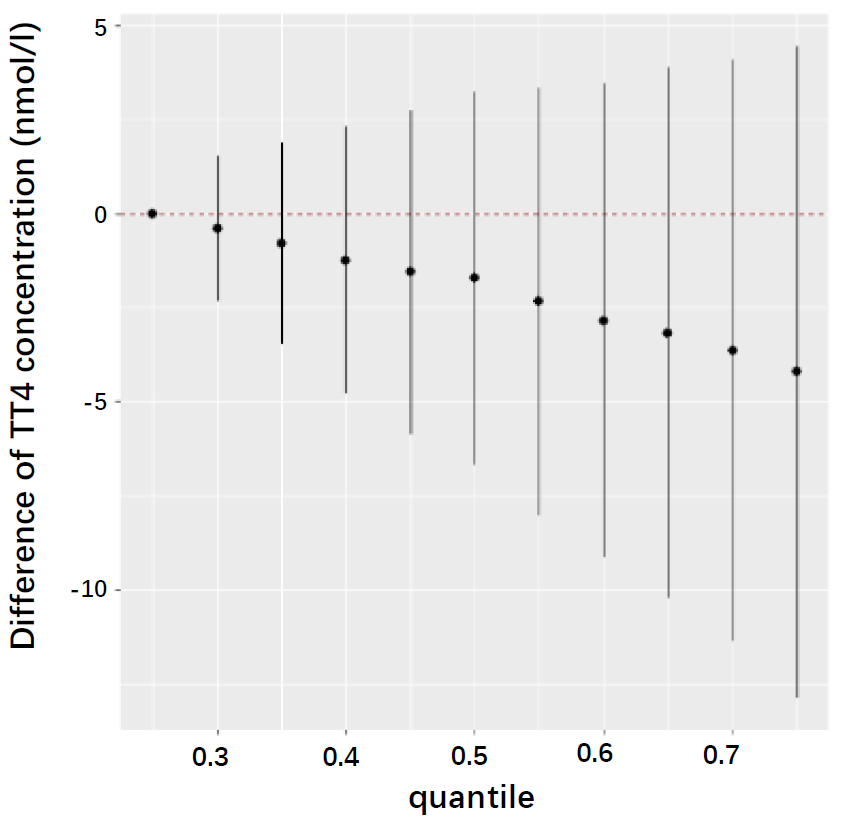

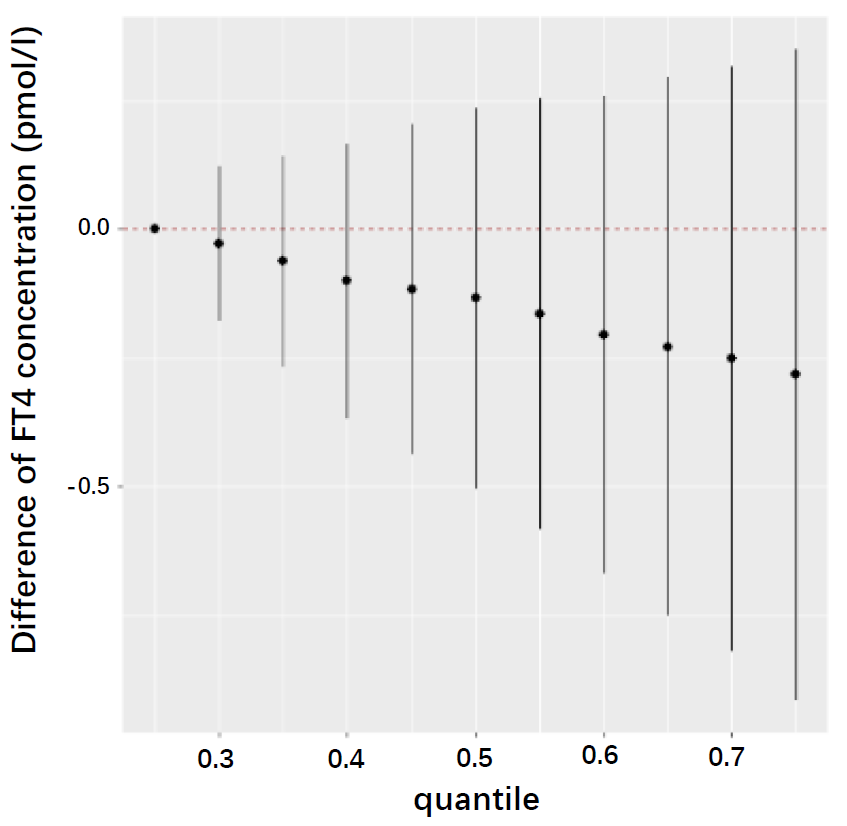

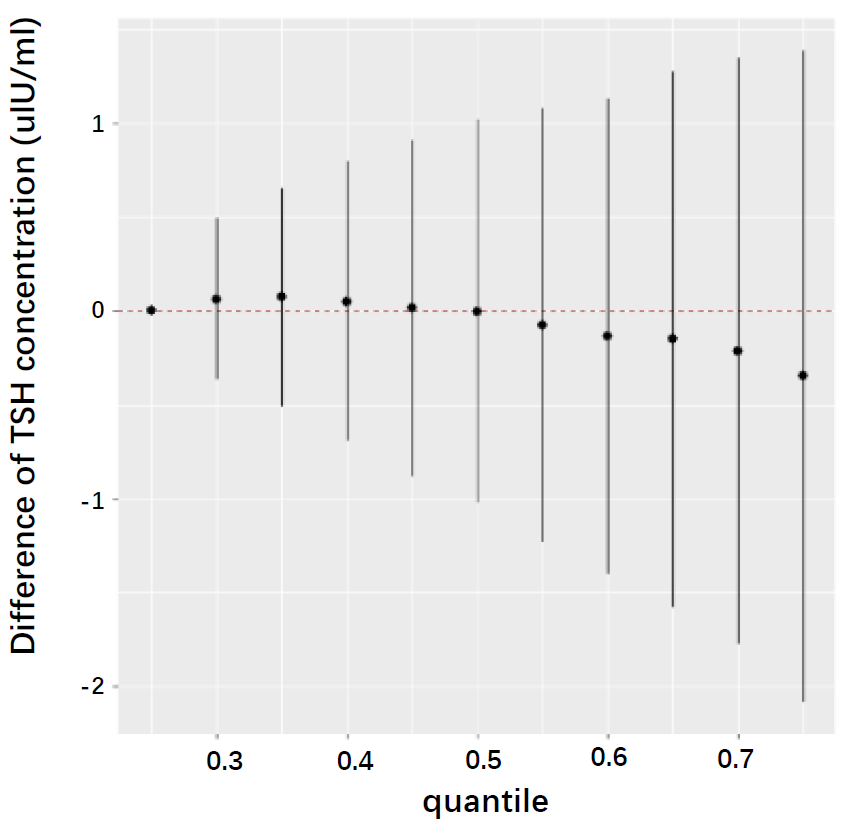


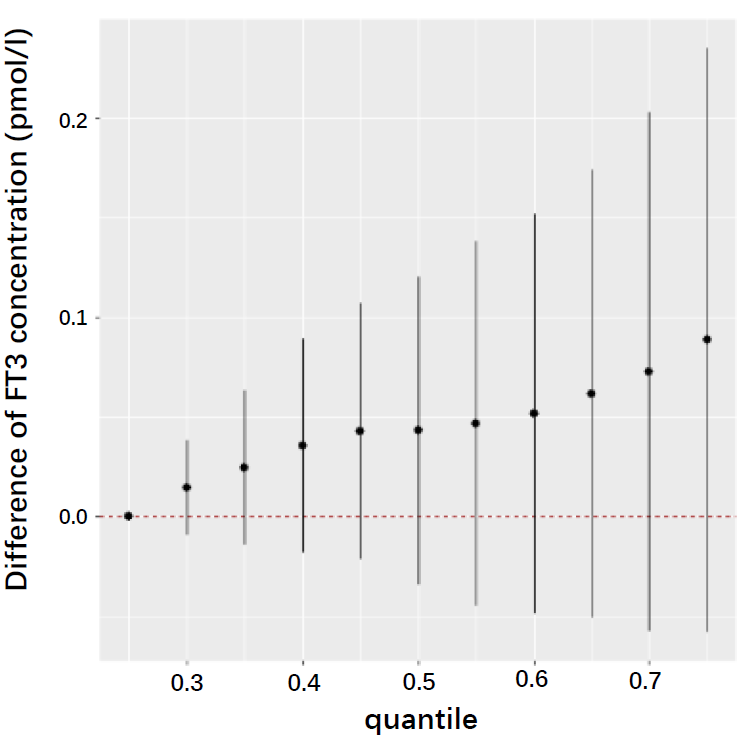

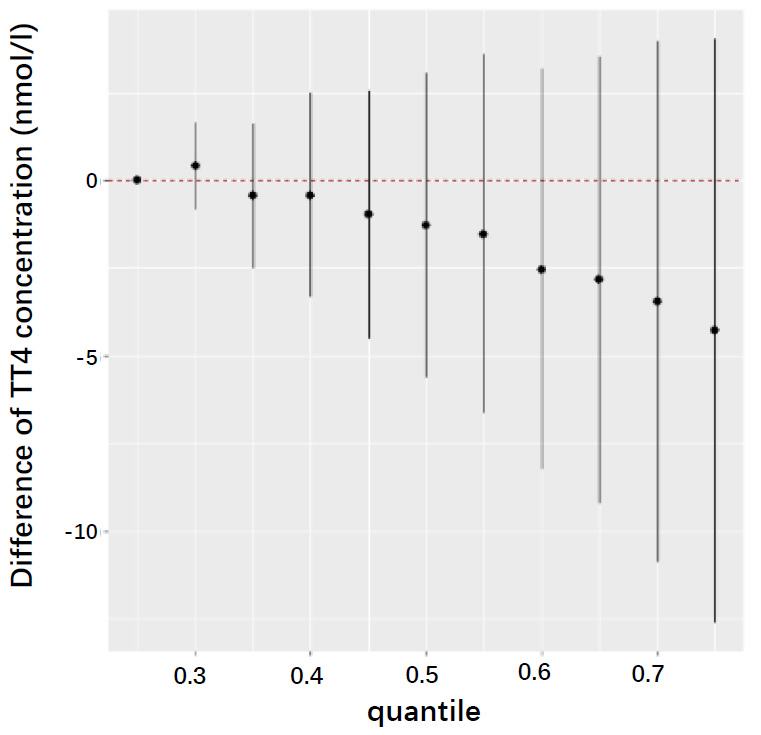

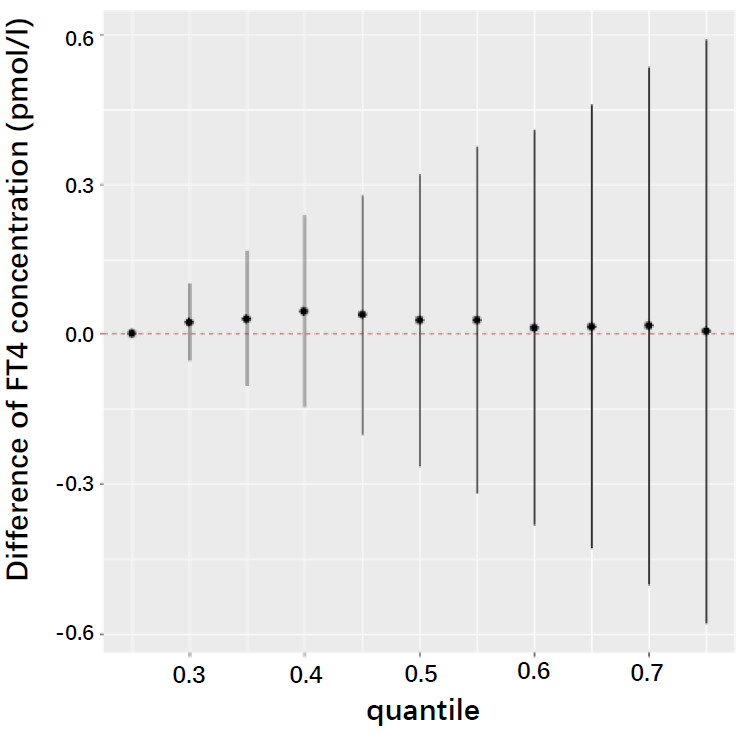

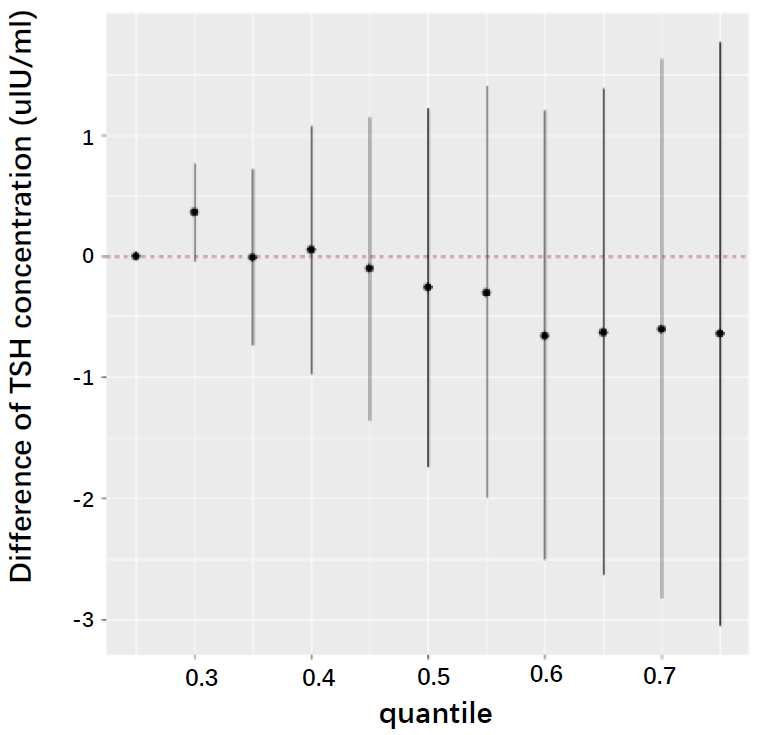


**B: Overall effect in girls**

**A: Overall effect in boys**

Note: The figure plots the estimated change in TH concentrations when all PFAS concentrations are fixed at specified quantiles (ranging from 0.25 to 0.75), as compared to when their concentrations are fixed at the 25^th^ percentile. Dots indicate the estimate, and vertical lines indicate the 95% credible intervals (CrI).

All models were adjusted for maternal age at delivery, pre-pregnancy BMI, education, parity, gestational age, delivery type, maternal passive smoking during pregnancy, maternal folic acid supplement, and paternal drinking during three months before pregnancy.

**Figure S5** Single-exposure effects of each individual PFAS on thyroid hormone concentrations in cord blood in Bayesian kernel machine regression models stratified by infant sex (A: in boys; B: in girls).


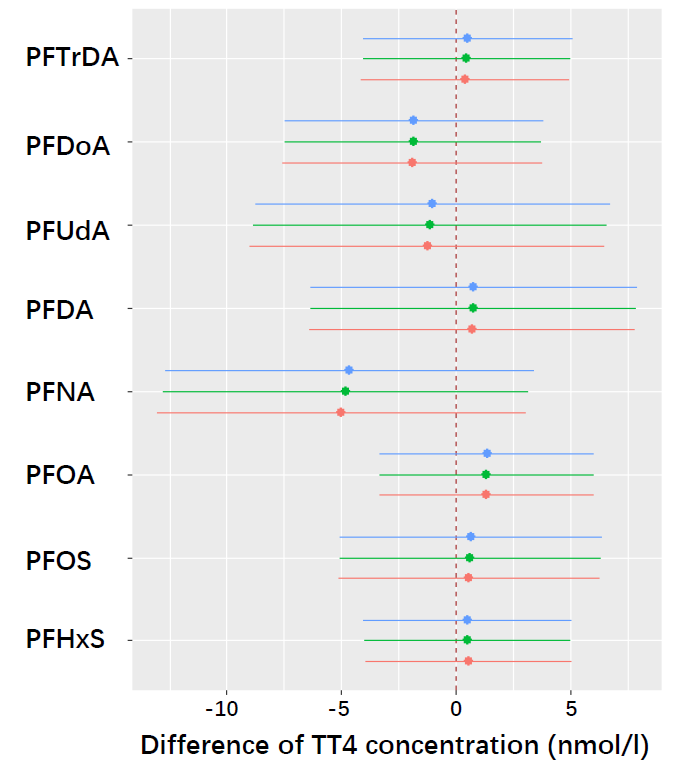

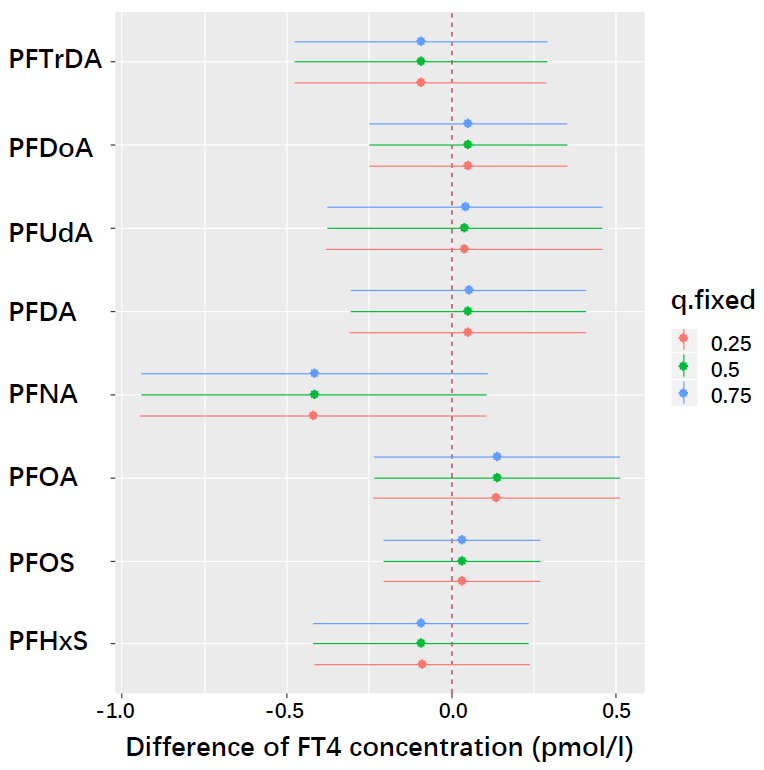


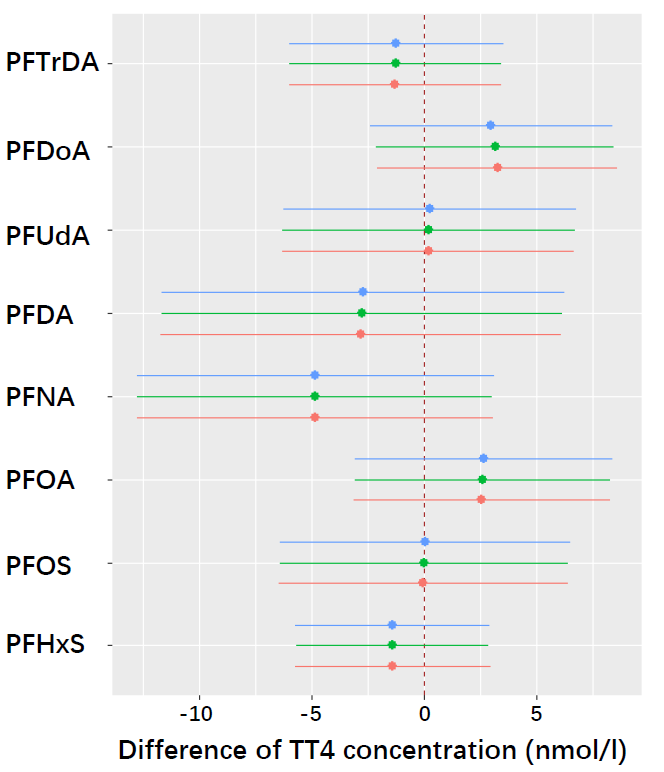

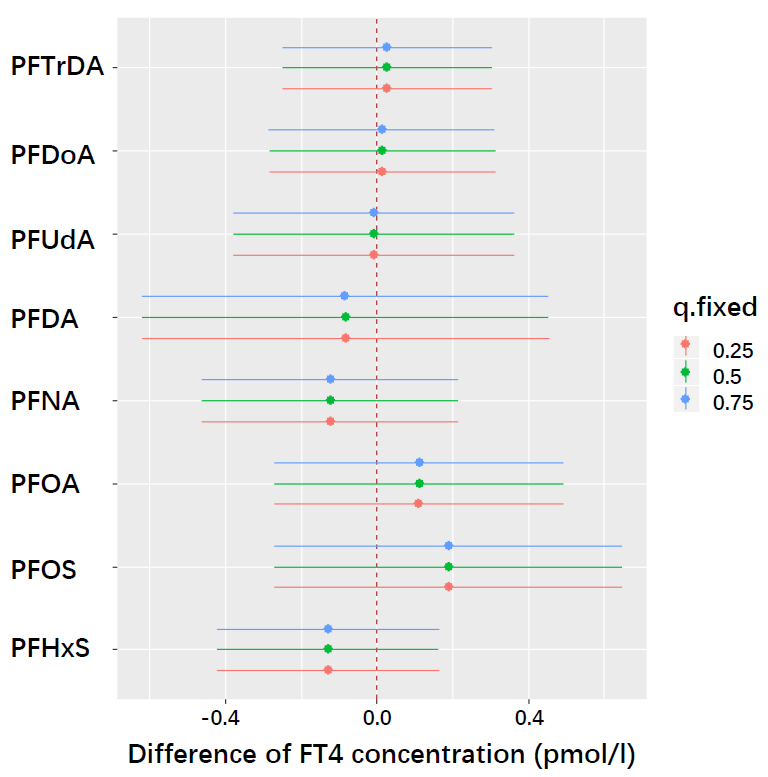


**A: Single-exposure effects among boys**

**B: Single-exposure effects among girls**

FT4

T4

FT4

T4

Note: This plot describes the change in hormone concentrations associated with a change in each individual PFAS from its 25^th^ to its 75^th^ percentile, when all the other PFASs are ﬁxed at either the 25^th^ (red line), 50^th^ (green line), or 75^th^ percentile (blue line). Dots indicate the estimate, and horizontal lines indicate the 95% credible intervals (CrI).

All models were adjusted for maternal age at delivery, pre-pregnancy BMI, education, parity, gestational age, delivery type, maternal passive smoking during pregnancy, maternal folic acid supplement, and paternal drinking during three months before pregnancy.

**Figure S6** Overall effects of the mixture of eight PFASs on thyroid hormones concentrations in cord blood in Bayesian kernel machine regression models stratified by type of delivery. A: T3 in infants with vaginal delivery (A1) and with caesarean section (A2); B: FT3 in infants with vaginal delivery (B1) and with caesarean section (B2); C: T4 in infants with vaginal delivery (C1) and with caesarean section (C2); D: FT4 in infants with vaginal delivery (D1) and with caesarean section (D2); E: TSH in infants with vaginal delivery (E1) and with caesarean section (E2).

D2

C2

B2

A2

E2

E1

D1

C1

B1

A1


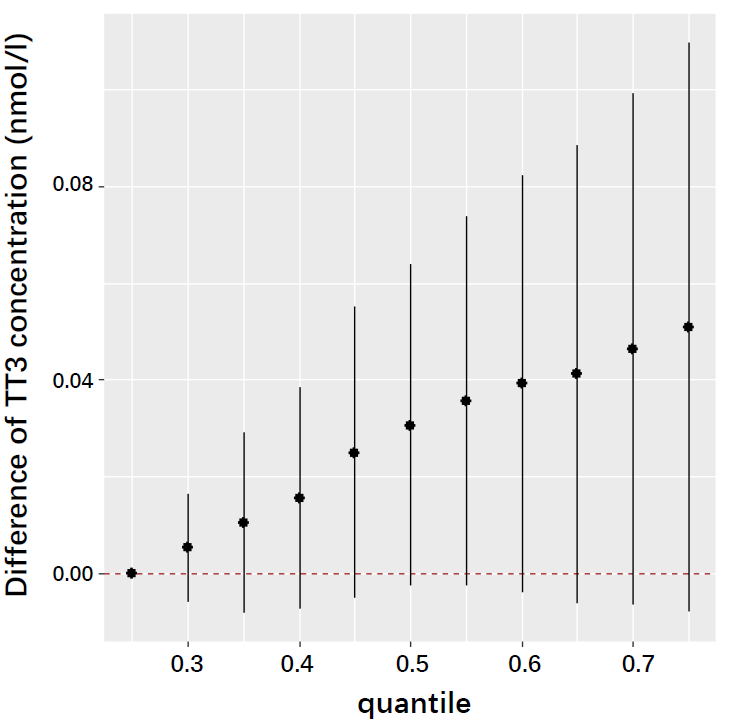

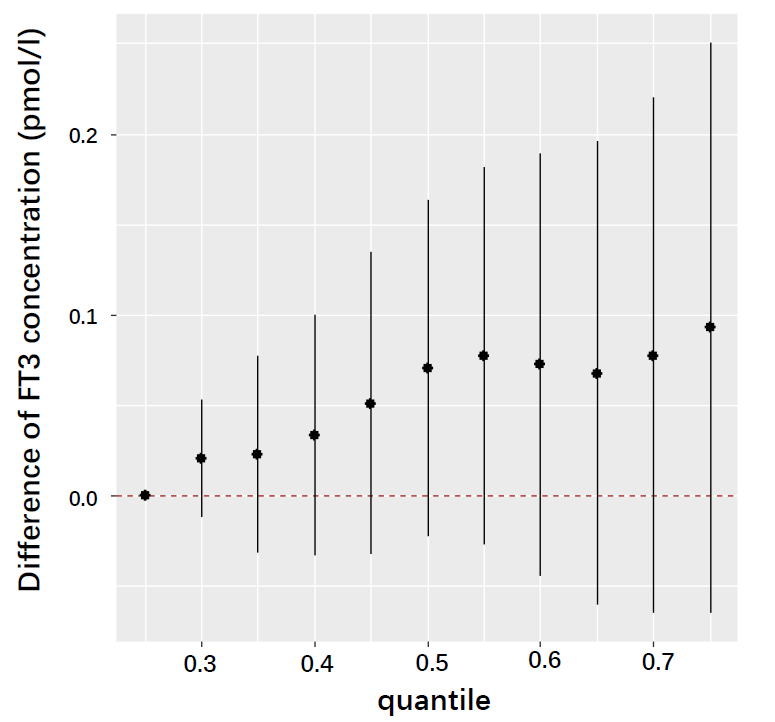

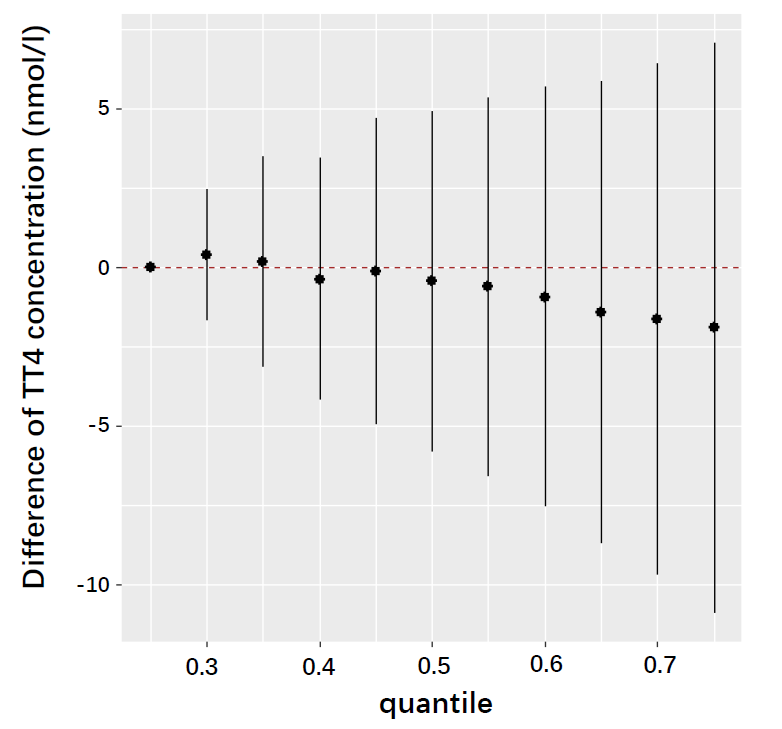

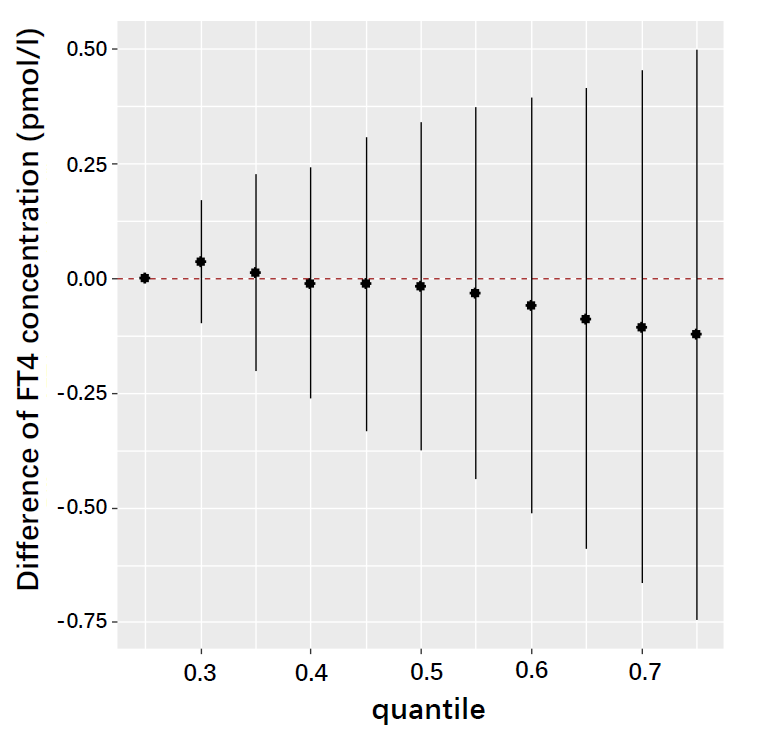

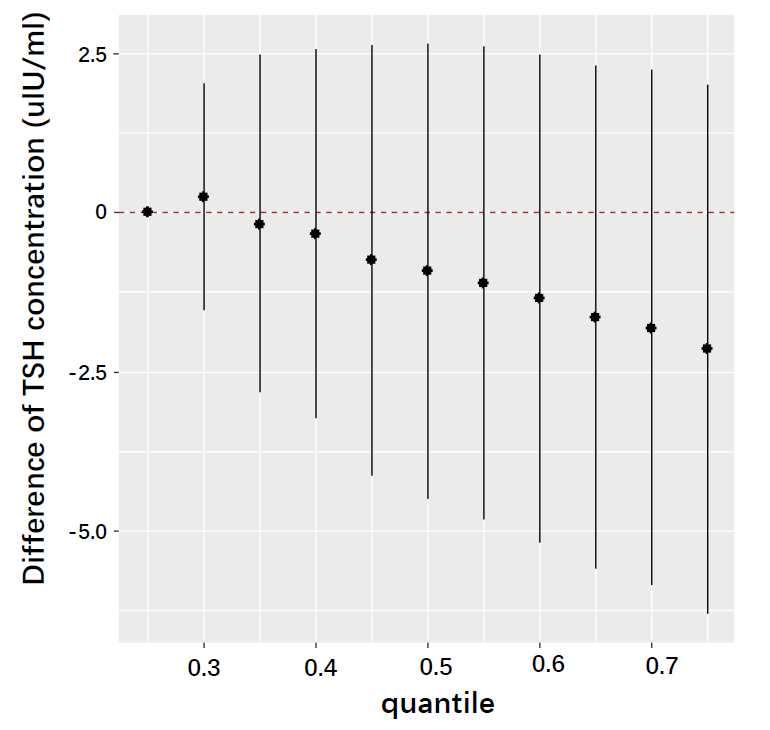

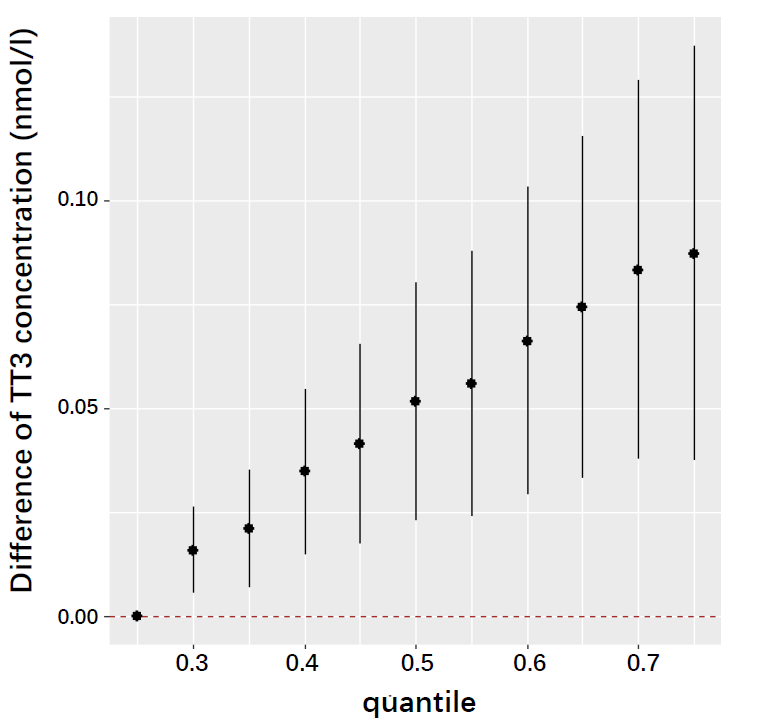

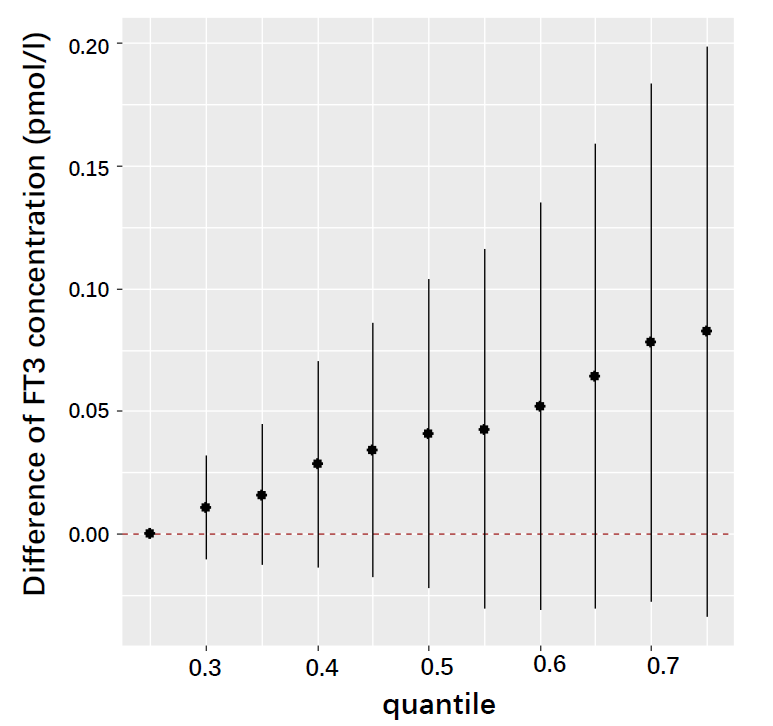

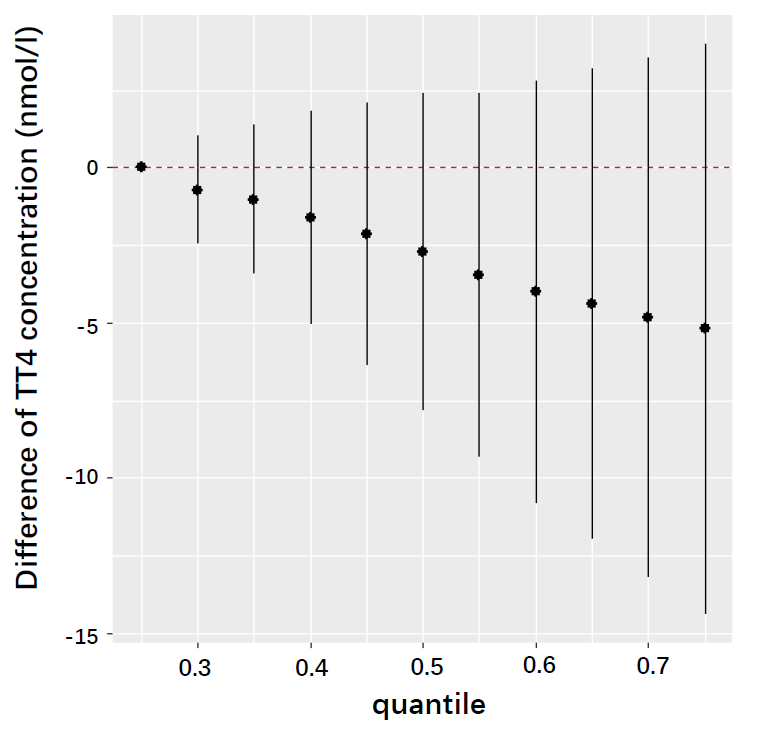

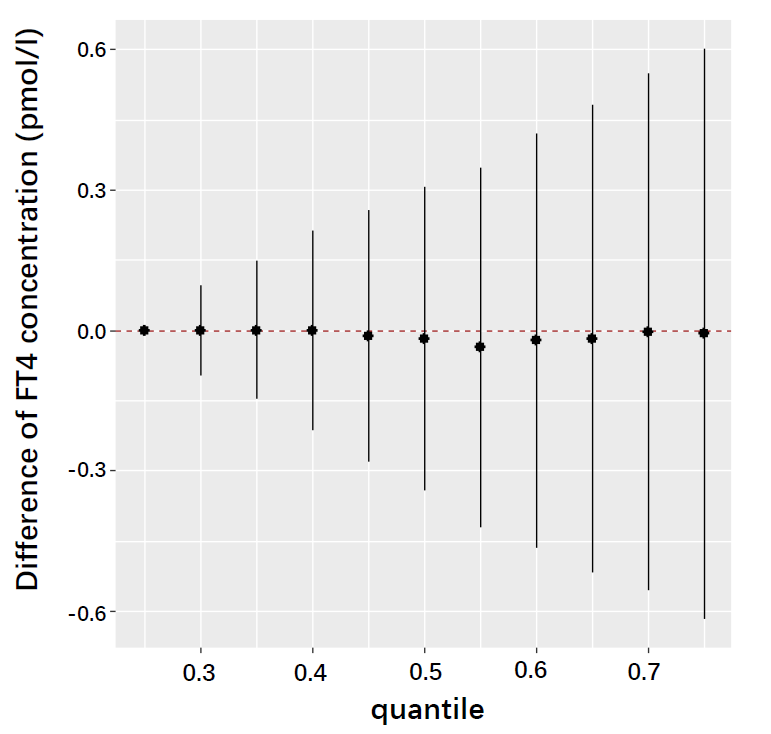

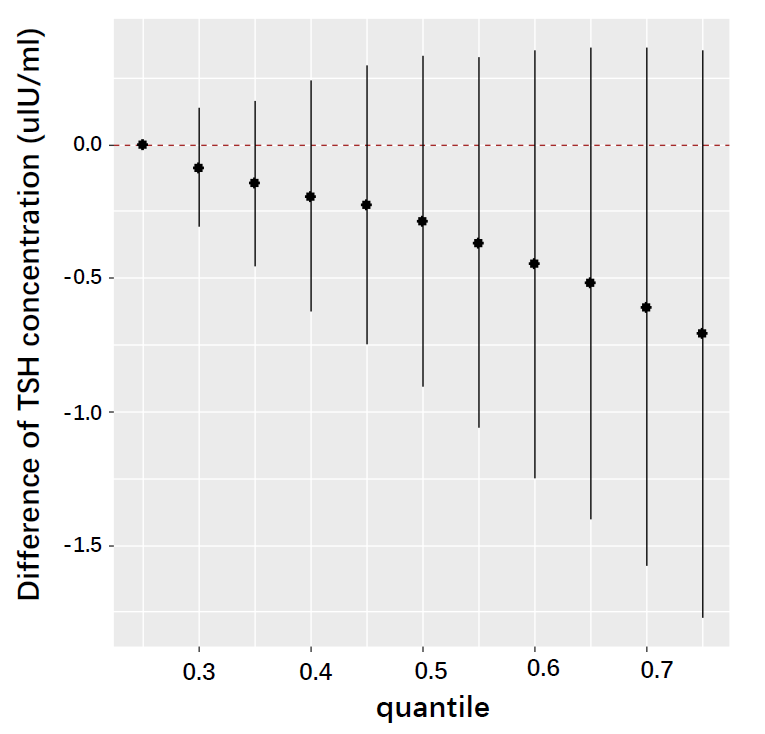


Note: The figure plots the estimated change in TH concentrations when all PFAS concentrations are fixed at specified quantiles (ranging from 0.25 to 0.75), as compared to when their concentrations are fixed at the 25^th^ percentile. Dots indicate the estimate, and vertical lines indicate the 95% credible intervals (CrI).

All models were adjusted for maternal age at delivery, pre-pregnancy BMI, education, parity, gestational age, infant sex, maternal passive smoking during pregnancy, maternal folic acid supplement, and paternal drinking during three months before pregnancy.

**Figure S7** Single-exposure effects of each individual PFAS on thyroid hormone concentrations in cord blood in Bayesian kernel machine regression models stratified by type of delivery. A: T3 in infants with vaginal delivery (A1) and with caesarean section (A2); B: FT3 in infants with vaginal delivery (B1) and with caesarean section (B2); C: T4 in infants with vaginal delivery (C1) and with caesarean section (C2); D: FT4 in infants with vaginal delivery (D1) and with caesarean section (D2); E: TSH in infants with vaginal delivery (E1) and with caesarean section (E2).

A2

B2

C2

D2

E2

E1

D1

C1

B1

A1


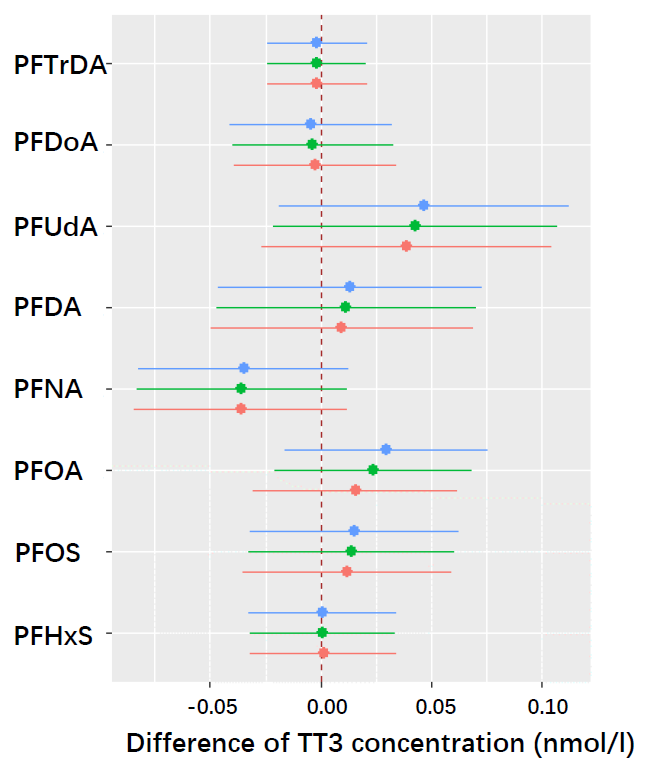

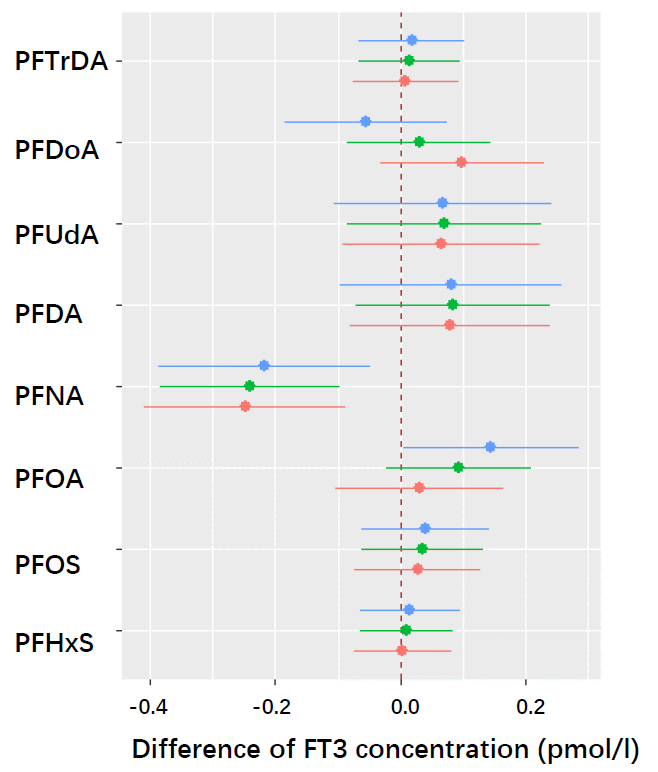

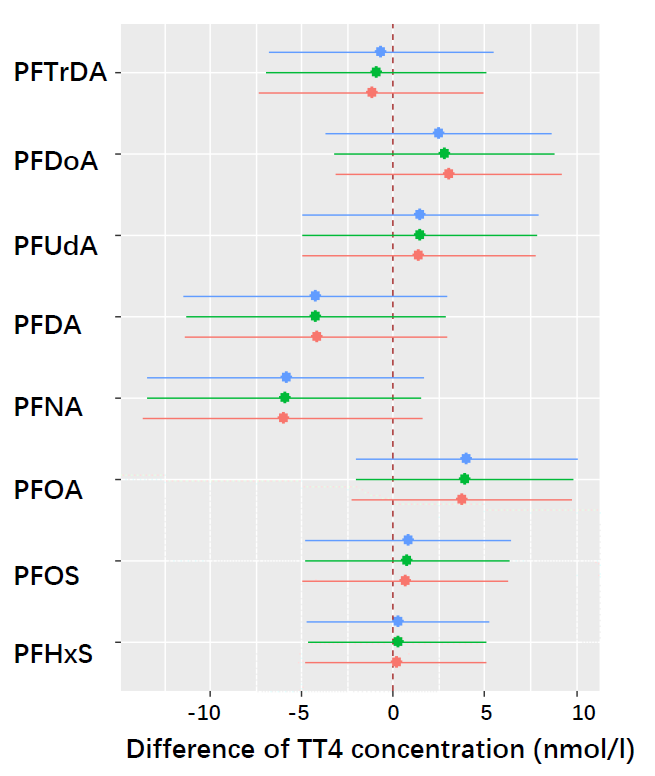

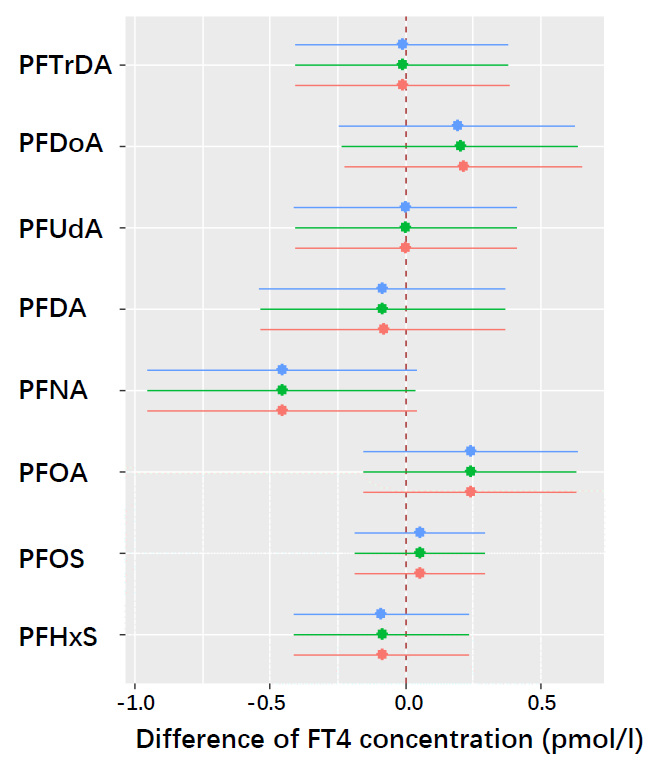

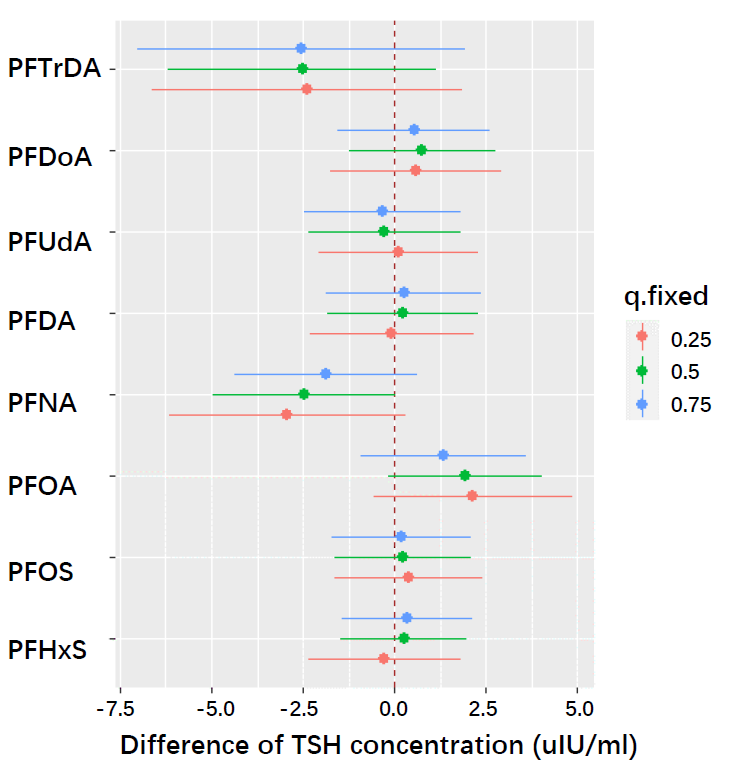

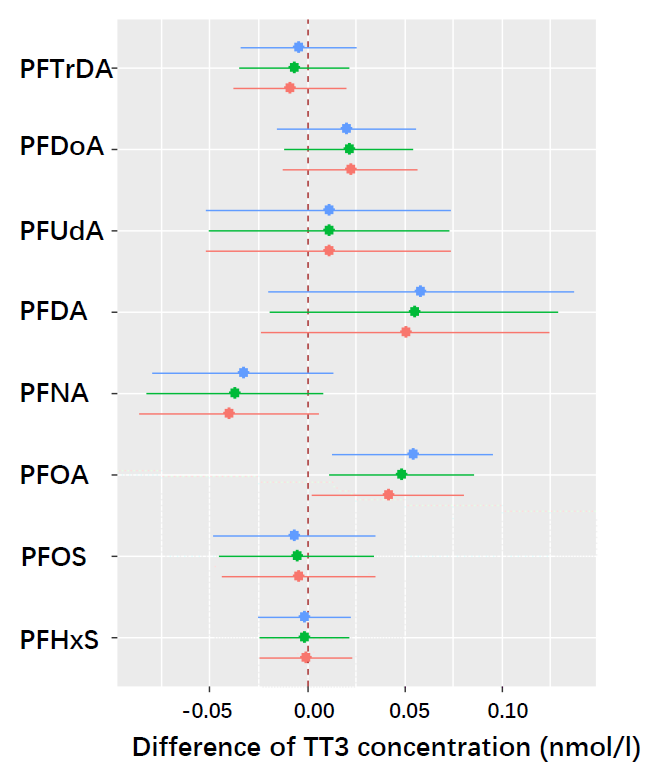

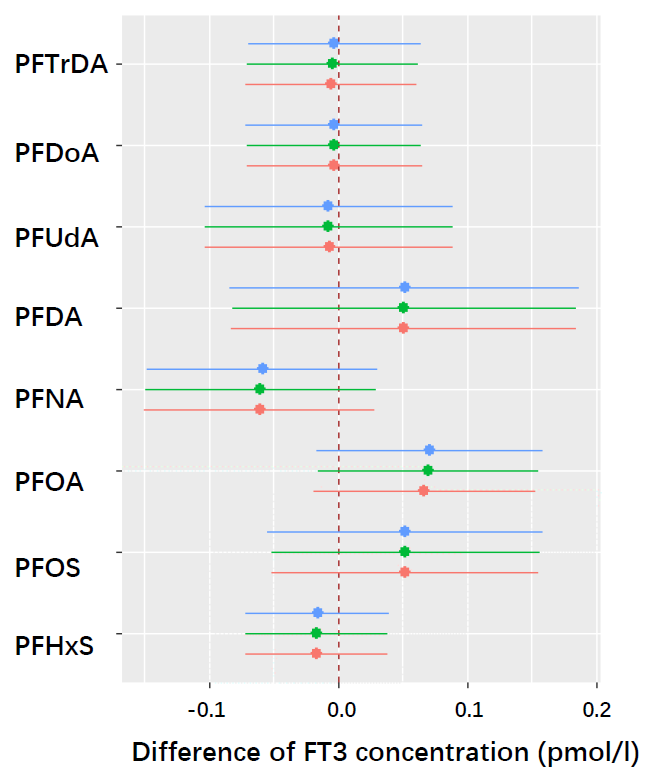

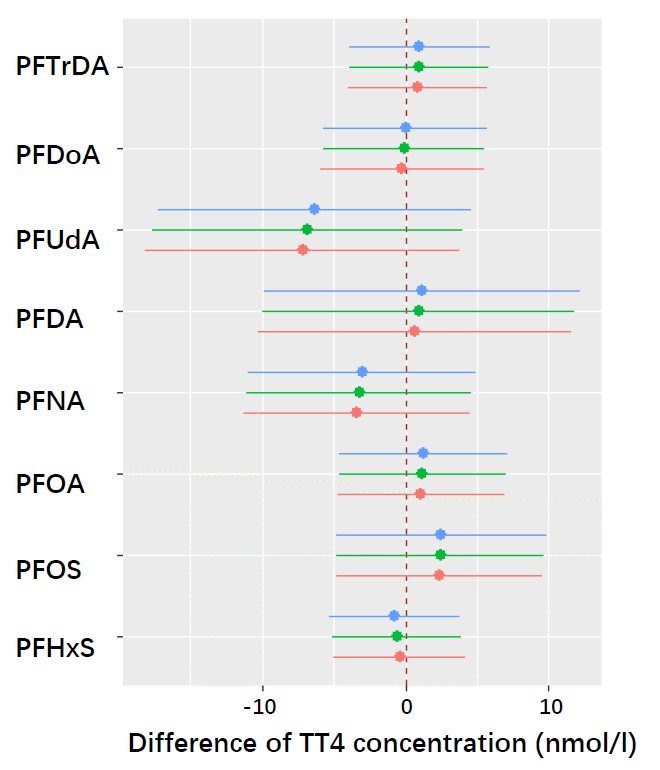

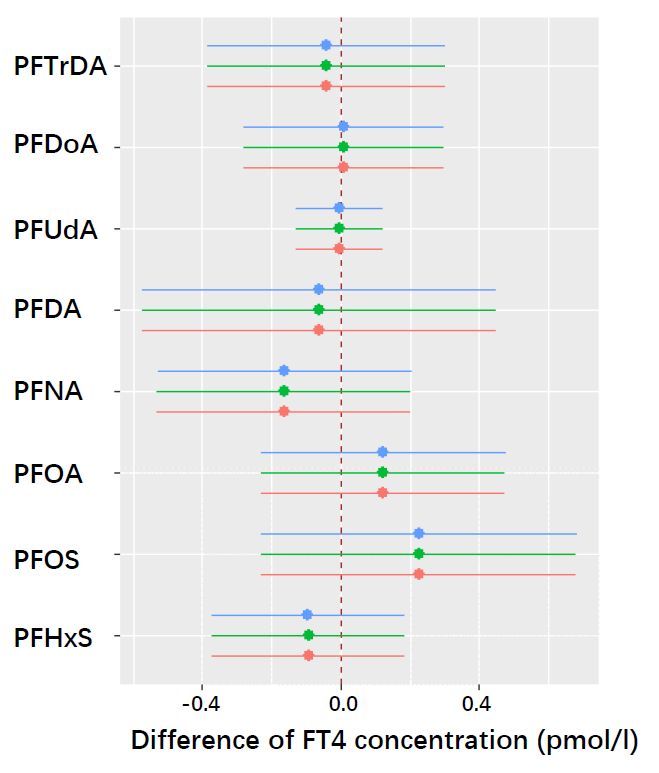

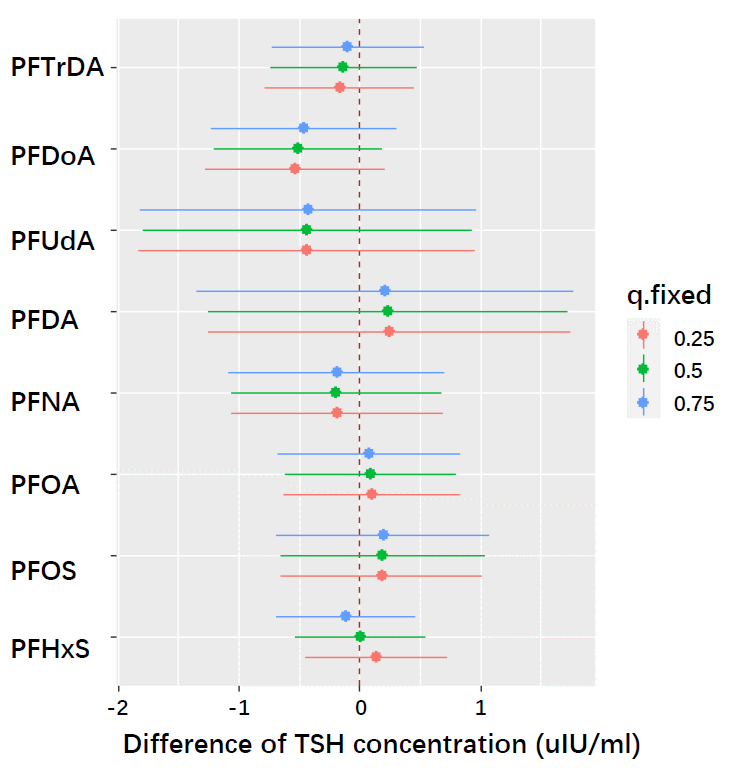


Note: This plot describes the change in cord plasma TH concentrations associated with a change in each individual PFAS from its 25^th^ to its 75^th^ percentile, when all the other PFASs are ﬁxed at either the 25^th^ (red line), 50^th^ (green line), or 75^th^ percentile (blue line). Dots indicate the estimate, and horizontal lines indicate the 95% credible intervals (CrI).

All models were adjusted for maternal age at delivery, pre-pregnancy BMI, education, parity, gestational age, infant sex, maternal passive smoking during pregnancy, maternal folic acid supplement, and paternal drinking during three months before pregnancy.

D

E

C

B

A


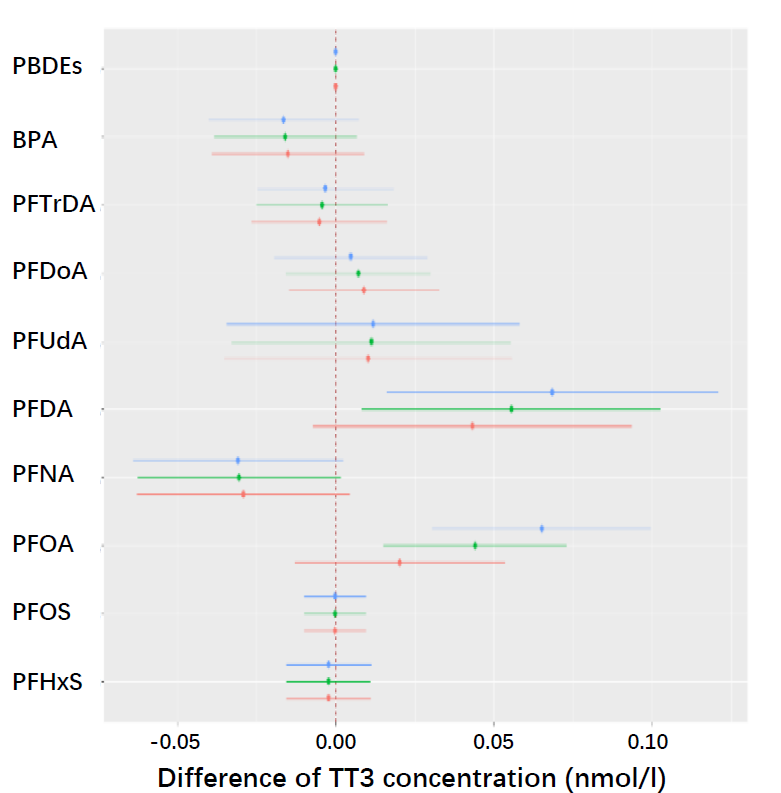

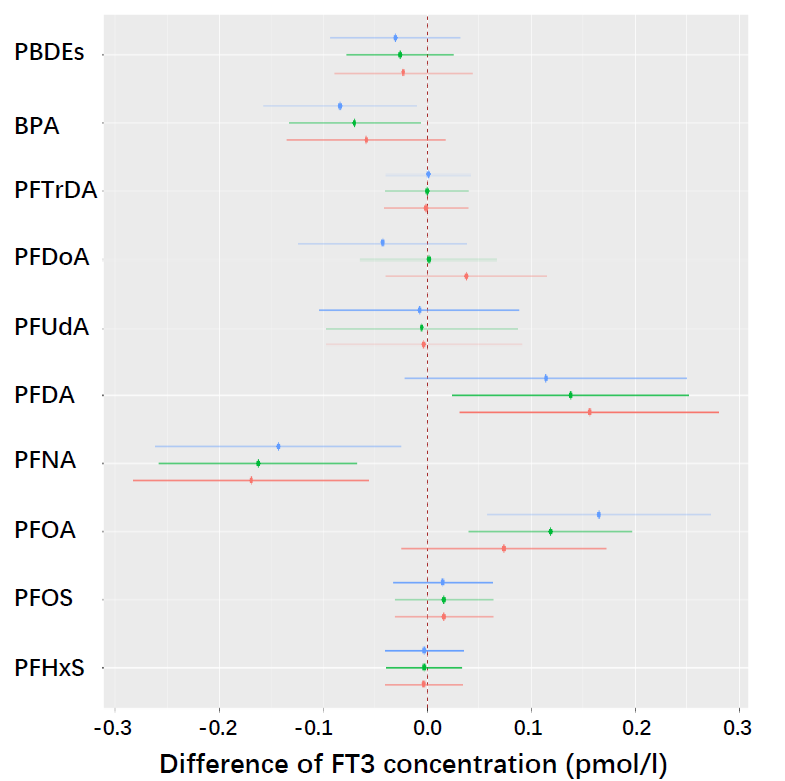

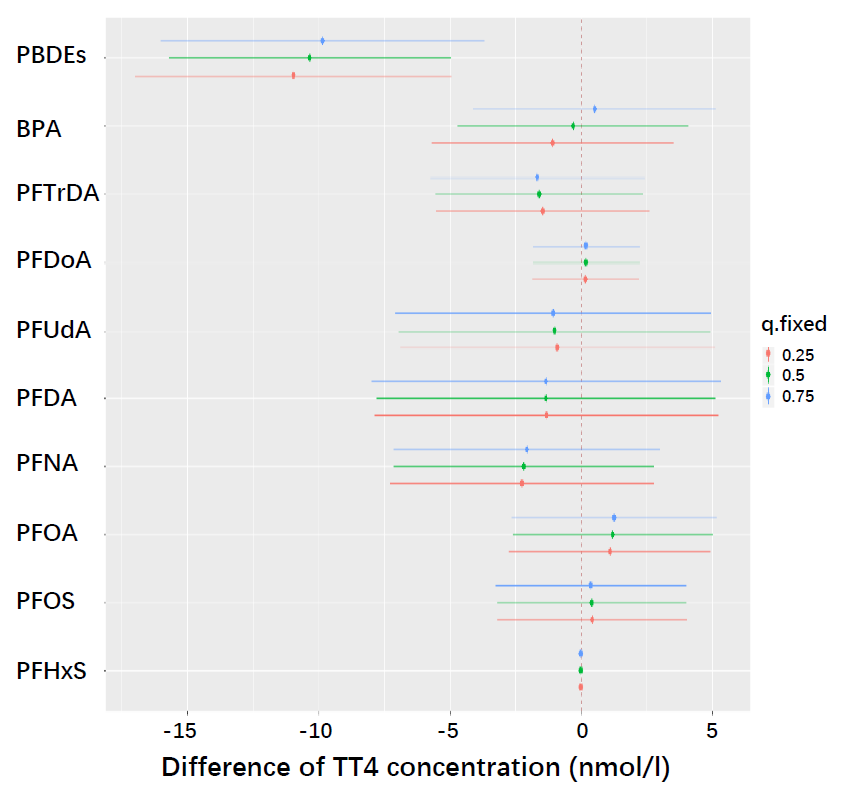

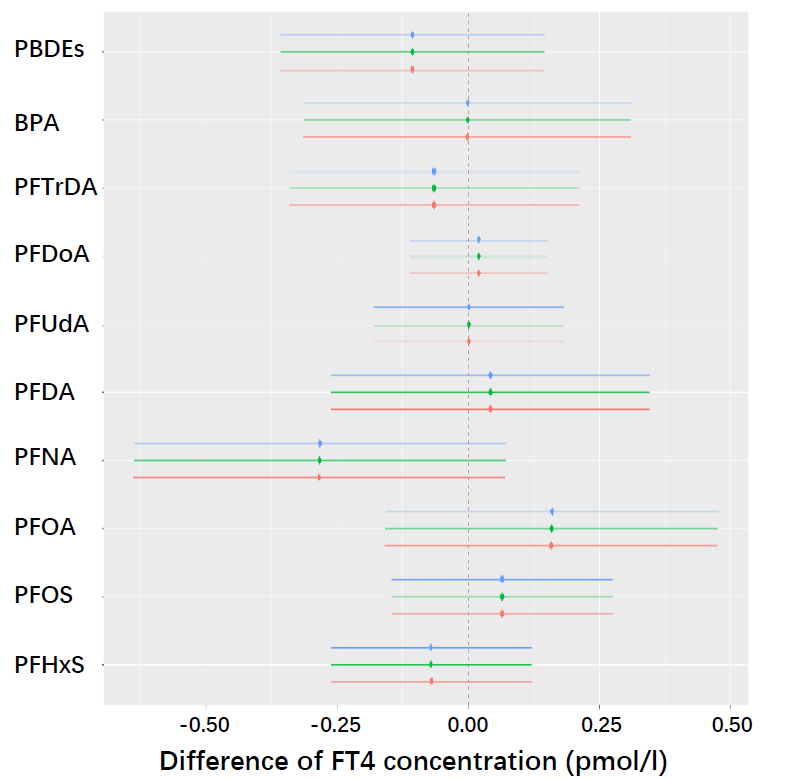

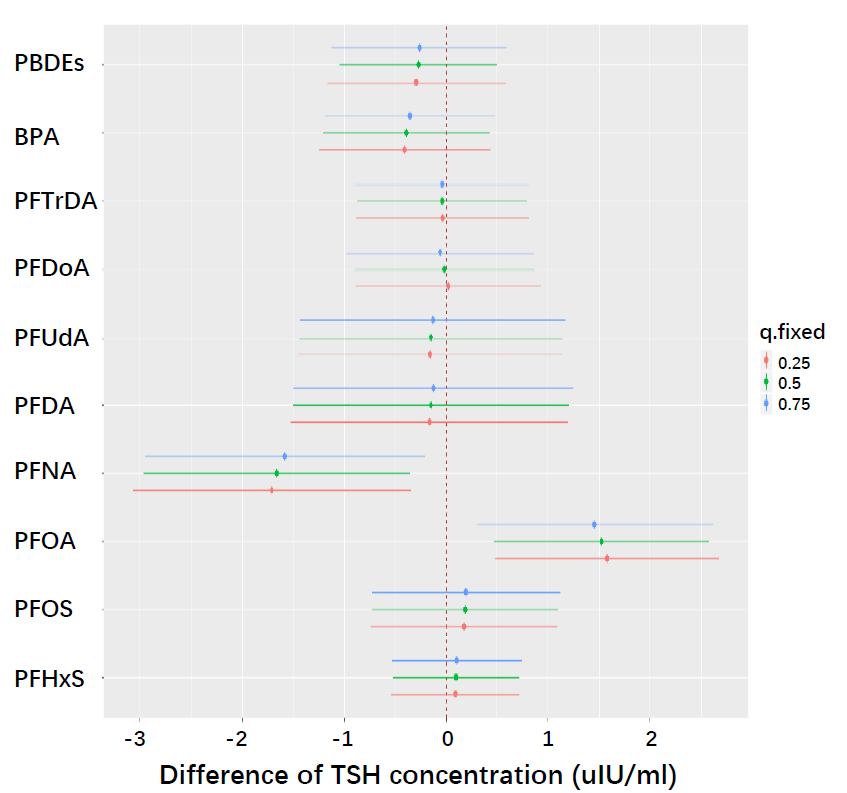


**Figure S8** Single-exposure effects of each individual PFAS, bisphenol A (BPA), and bolybrominated diphenyl ethers (Sum_5_PBDEs) on thyroid hormone concentrations in cord blood in Bayesian kernel machine regression models.

Note: This plot describes the change in hormone concentrations associated with a change in each individual PFAS, BPA, and Sum_5_PBDEs from its 25^th^ to its 75^th^ percentile, when all the other chemicals are ﬁxed at either the 25^th^ (red line), 50^th^ (green line), or 75^th^ percentile (blue line). Dots indicate the estimate, and horizontal lines indicate the 95% credible intervals (CrI). Sum_5_PBDEs, the sum of BDE-28, -47, -99, -100, and -153 concentrations.

All models were adjusted for maternal age at delivery, pre-pregnancy BMI, education, parity, gestational age, delivery type, infant sex, maternal passive smoking during pregnancy, maternal folic acid supplement, and paternal drinking during three months before pregnancy.

**Table S1** Comparisons of maternal plasma PFAS concentrations between mother-infant pairs included and excluded from the present study using Mann–Whitney U test

| PFAS(ng/mL) | Included (N = 300) |  | Excluded (N = 679) | Z^#^ | *p*-value |
| --- | --- | --- | --- | --- | --- |
|  | Median (Q1, Q3) * |  | Median (Q1, Q3) |  |  |
| PFHxS | 2.67(2.04, 3.40) | | 2.78(2.12, 3.60) | -1.3572 | 0.1747 |
| PFOS | 10.49(7.34, 16.30) | | 10.9(7.66, 15.59) | -0.3735 | 0.7088 |
| PFOA | 19.38(14.49, 26.80) | | 20.19(15.53, 26.91) | -0.9122 | 0.3617 |
| PFNA | 1.8(1.38, 2.51) | | 1.76(1.27, 2.49) | 0.9522 | 0.3410 |
| PFDA | 2.15(1.41, 3.28) | | 2.01(1.23, 3.25) | 1.5016 | 0.1332 |
| PFUdA | 1.68(1.04, 2.55) | | 1.51(0.95, 2.47) | 1.1731 | 0.2408 |
| PFDoA | 0.11(0.05, 0.20) | | 0.12(0.06, 0.21) | -1.7626 | 0.0780 |
| PFTrDA | 0.11(0.04, 0.18) | | 0.11(0.05, 0.20) | -0.9806 | 0.3268 |

*Q1: the first quartile; Q3: the third quartile.

^#^ Statistic of Mann-Whitney U test.

**Table S2.** Pearson correlation coeﬃcients between pairs of ln-transformed PFAS concentrations (ng/mL) in maternal plasma

|  | **ln_PFHxS** | **ln_PFOS** | **ln_PFOA** | **ln_PFNA** | **ln_PFDA** | **ln_PFUdA** | **ln_PFDoA** | **ln_PFTrDA** |
| --- | --- | --- | --- | --- | --- | --- | --- | --- |
| **ln_PFHxS** | 1 |  |  |  |  |  |  |  |
| **ln_PFOS** | 0.14 | 1 |  |  |  |  |  |  |
| **ln_PFOA** | 0.12 | 0.36* | 1 |  |  |  |  |  |
| **ln_PFNA** | 0.06 | 0.64* | 0.59* | 1 |  |  |  |  |
| **ln_PFDA** | 0.05 | 0.73* | 0.43* | 0.75* | 1 |  |  |  |
| **ln_PFUdA** | 0.02 | 0.72* | 0.34* | 0.72* | 0.89* | 1 |  |  |
| **ln_PFDoA** | 0.07 | 0.40* | 0.15 | 0.44* | 0.54* | 0.54* | 1 |  |
| **ln_PFTrDA** | -0.04 | 0.35* | 0.09 | 0.35* | 0.40* | 0.44* | 0.33* | 1 |

* *P* < 0.0001

**Table S3.** Pearson correlation coeﬃcients between pairs of thyroid hormones in 300 cord plasma samples

|  | **TSH** | **T4** | **FT4** | **FT3** | **T3** |
| --- | --- | --- | --- | --- | --- |
| **TSH** (μIU/ml) | 1.00 |  |  |  |  |
| **T4** (nmol/l) | 0.39* | 1.00 |  |  |  |
| **FT4** (pmol/l) | 0.24* | 0.82* | 1.00 |  |  |
| **FT3** (pmol/l) | 0.14 | 0.44* | 0.49* | 1.00 |  |
| **T3** (nmol/l) | -0.04 | 0.16 | 0.21 | 0.79* | 1.00 |

T_3_, total triiodothyronine; T_4_, total thyroxine; FT_3_, free triiodothyronine; FT_4_, free thyroxin; TSH, thyroid stimulating hormone.

* *P*-value < 0.0001

**Table S4.** Thyroid hormone concentrations in cord blood among infants born via vaginal delivery or caesarean section.

| Variables | Participants included (n=300) | T3  (nmol/l) | FT3  (pmol/l) | T4  (nmol/l) | FT4  (pmol/l) | TSH  (μIU/ml) | |
| --- | --- | --- | --- | --- | --- | --- | --- |
| Type of delivery | | | | | | | |
| Vaginal | 154 (51.7) | 0.8±0.2 | **1.8±0.4*** | 94.4±26.6 | **14.0±1.9*** | **7.7(5.3,11.7)*** |  |
| Cesarean section | 144 (48.3) | 0.9±0.1 | **1.9±0.3*** | 94.9±27.5 | **14.5±2.0*** | **5.7(3.9,7.2)*** |  |

*Student’s t-test, P < 0.05

**Table S5**. Associations between maternal plasma PFAS concentrations (tertile, ng/mL) and thyroid hormones in cord plasma by linear regression models (n=280)*.

|  |  |  | β (95%CI) |  |  |
| --- | --- | --- | --- | --- | --- |
|  | **T3**  (nmol/l) | **FT3**  (pmol/l) | **T4**  (nmol/l) | **FT4**  (pmol/l) | **TSH**  (μIU/ml) |
| PFHxS |  |  |  |  |  |
| Low tertile | Ref | Ref | Ref | Ref | Ref |
| Middle tertile | **0.05 (0.00, 0.09)*** | 0.08 (-0.02, 0.19) | -1.06 (-8.33, 6.20) | -0.02 (-0.56, 0.52) | 0.02 (-1.42, 1.45) |
| High tertile | 0.01 (-0.04, 0.05) | 0.05 (-0.05, 0.16) | -2.27 (-9.90, 5.35) | ***-0.50 (-1.07, 0.06)^#^*** | 0.43 (-1.07, 1.93) |
| *P* for trend | 0.6279 | 0.3441 | 0.5366 | ***0.0898*** | 0.6971 |
| PFOS |  |  |  |  |  |
| Low tertile | Ref | Ref | Ref | Ref | Ref |
| Middle tertile | **0.08 (0.04, 0.13) *** | **0.13 (0.02, 0.23) *** | ***-7.09 (-14.54, 0.35) ^#^*** | -0.25 (-0.80, 0.31) | -1.06 (-2.53, 0.41) |
| High tertile | **0.08 (0.03, 0.12) *** | **0.11 (0.01, 0.22) *** | -4.58 (-11.96, 2.81) | 0.20 (-0.35, 0.75) | -1.19 (-2.65, 0.27) |
| *P* for trend | **0.0010** | **0.0467** | 0.1835 | 0.4902 | ***0.0942*** |
| PFOA |  |  |  |  |  |
| Low tertile | Ref | Ref | Ref | Ref | Ref |
| Middle tertile | -0.01 (-0.05, 0.04) | -0.05 (-0.16, 0.05) | **-12.72 (-20.31, -5.12) *** | **-0.79 (-1.36, -0.22) *** | -1.11 (-2.62, 0.4) |
| High tertile | **0.07 (0.03, 0.12) *** | **0.12 (0.02, 0.23) *** | -0.27 (-7.50, 6.97) | 0.13 (-0.41, 0.67) | 1.09 (-0.35, 2.52) |
| *P* for trend | **0.0020** | **0.0316** | 0.9331 | 0.6767 | 0.1415 |
| PFNA |  |  |  |  |  |
| Low tertile | Ref | Ref | Ref | Ref | Ref |
| Middle tertile | 0.02 (-0.03, 0.07) | -0.04 (-0.15, 0.07) | -2.57 (-10.19, 5.06) | 0.03 (-0.54, 0.59) | ***-1.33 (-2.82, 0.17) ^#^*** |
| High tertile | **0.06 (0.01, 0.10) *** | -0.01 (-0.13, 0.10) | **-9.64 (-17.45, -1.84) *** | ***-0.55 (-1.13, 0.03) ^#^*** | **-2.57 (-4.10, -1.05) *** |
| *P* for trend | **0.0321** | 0.8023 | **0.0214** | ***0.0859*** | **0.0027** |
| PFDA |  |  |  |  |  |
| Low tertile | Ref | Ref | Ref | Ref | Ref |
| Middle tertile | **0.06 (0.02, 0.11) *** | 0.09 (-0.02, 0.19) | -3.49 (-11.03, 4.05) | 0.10 (-0.47, 0.66) | 0.13 (-1.36, 1.62) |
| High tertile | **0.10 (0.06, 0.15) *** | **0.13 (0.02, 0.24) *** | **-10.02 (-17.66, -2.38) *** | -0.26 (-0.84, 0.31) | ***-1.29 (-2.80, 0.22) ^#^*** |
| *P* for trend | **<.0001** | **0.0288** | **0.0137** | 0.4177 | 0.1041 |
| PFUdA |  |  |  |  |  |
| Low tertile | Ref | Ref | Ref | Ref | Ref |
| Middle tertile | **0.07 (0.02, 0.11) *** | 0.09 (-0.02, 0.20) | -1.33 (-8.89, 6.24) | 0.10 (-0.46, 0.67) | -1.08 (-2.56, 0.41) |
| High tertile | **0.07 (0.02, 0.12) *** | 0.06 (-0.05, 0.17) | ***-6.47 (-14.04, 1.09) ^#^*** | -0.20 (-0.76, 0.37) | **-1.92 (-3.40, -0.43) *** |
| *P* for trend | **0.0048** | 0.3342 | 0.1112 | 0.5742 | **0.0191** |
| PFDoA |  |  |  |  |  |
| Low tertile | Ref | Ref | Ref | Ref | Ref |
| Middle tertile | **0.07 (0.02, 0.11) *** | **0.16 (0.05, 0.26) *** | 1.59 (-5.92, 9.10) | 0.45 (-0.11, 1.01) | -1.13 (-2.60, 0.35) |
| High tertile | ***0.04 (-0.01, 0.08) ^#^*** | -0.01 (-0.11, 0.10) | -4.15 (-11.48, 3.19) | 0.05 (-0.49, 0.60) | ***-1.37 (-2.81, 0.07) ^#^*** |
| *P* for trend | **0.0746** | 0.9255 | 0.3807 | 0.6488 | **0.0836** |
| PFTrDA |  |  |  |  |  |
| Low tertile | Ref | Ref | Ref | Ref | Ref |
| Middle tertile | 0.01 (-0.04, 0.05) | 0.03 (-0.07, 0.14) | -1.11 (-8.55, 6.33) | 0.14 (-0.42, 0.69) | -0.23 (-1.69, 1.23) |
| High tertile | 0.02 (-0.03, 0.06) | -0.01 (-0.12, 0.10) | -4.93 (-12.60, 2.74) | -0.27 (-0.84, 0.30) | **-1.56 (-3.06, -0.06) *** |
| *P* for trend | 0.5279 | 0.7303 | 0.1738 | 0.3341 | **0.0405** |

Adjusted for maternal age at delivery, pre-pregnancy BMI, education, parity, gestational age, delivery type, and infant sex.

* P<0.05; ^#^ 0.05<P<0.1.
